# Epidemiologic characteristics of early cases with 2019 novel coronavirus (2019-nCoV) disease in Korea

**DOI:** 10.4178/epih.e2020007

**Published:** 2020-02-09

**Authors:** Moran Ki, Task Force for 2019-nCoV

**Affiliations:** 1 Department of Cancer Control and Population Health, Graduate School of Cancer Science and Policy, National Cancer Center, Goyang, Korea

**Keywords:** 2019-nCoV, Quarantine, Isolation, Outbreak, Epidemiology, Korea

## Abstract

In about 20 days since the diagnosis of the first case of the 2019 novel coronavirus (2019-nCoV) in Korea on January 20, 2020, 28 cases have been confirmed. Fifteen patients (53.6%) of them were male and median age of was 42 years (range, 20-73). Of the confirmed cases, 16, 9, and 3 were index (57.2%), first-generation (32.1%), and second-generation (10.7%) cases, respectively. All first-generation and second-generation patients were family members or intimate acquaintances of the index cases with close contacts. Fifteen among 16 index patients had entered Korea from January 19 to 24, 2020 while 1 patient had entered Korea on January 31, 2020. The average incubation period was 3.9 days (median, 3.0), and the reproduction number was estimated as 0.48. Three of the confirmed patients were asymptomatic when they were diagnosed. Epidemiological indicators will be revised with the availability of additional data in the future. Sharing epidemiological information among researchers worldwide is essential for efficient preparation and response in tackling this new infectious disease.

## INTRODUCTION

On the last day of 2019, Chinese authorities officially announced that they were managing an outbreak of pneumonia with an unknown cause. The date of onset for the initial case of this new infectious disease remains unclear. According to a report published by the medical team in Wuhan, China, on the 41 cases of this infection that occurred between December 1, 2019 and January 2, 2020, the initial patient reportedly experienced symptom-onset on December 1, 2019, although he reportedly had never visited the Huanan Seafood Wholesale Market [1]. Therefore, it is likely that there was an unknown index patient, if this patient was infected by another person.

On January 7, 2020, the idiopathic pneumonia was reported to have been caused by a new coronavirus, and information regarding the organism was made available to researchers around the world [2]. The World Health Organization (WHO) tentatively named this new virus as the 2019 novel coronavirus (2019-nCoV). On January 10, 2020, the first death caused by this new infectious disease was reported in China. On January 13, the first confirmed case outside China was reported in Thailand, and the patient did not have a history of visiting the Huanan Seafood Wholesale Market. Thereafter, first cases were reported in Japan and Korea on January 15, 2020 and January 20, 2020, respectively. Since then, as of February 8, 2020, total number of confirmed cases in Korea have increased to 24 [3]. As epidemiologic characteristics of this new disease are unknown, they are being investigated based on comparisons with the clinically similar Severe Acute Respiratory Syndrome and the Middle East Respiratory Syndrome. Rapid investigation and determination of epidemiologic characteristics of new infectious diseases is crucial for limiting transmission and for attaining desirable treatment outcomes through early diagnosis and management. Sharing of crucial data, as unearthed by epidemiologists around the world, is highly critical and it could help to definitively determine characteristics of this new infectious disease and containing its additional spread accordingly is urgent.

This is a report reviewing the epidemiologic characteristics based on data of 28 patients in Korea between January 20, 2020 and February 10, 2020. The information may be revised on the basis of updated epidemiologic information.

## MATERIALS AND METHODS

This report was compiled using information from the epidemiologic investigation report by Korea Centers for Disease Control and Prevention (KCDC), and from additional data confirmed and announced by the press [3,4]. As dates of the onset of symptom were recorded based on patients’ statements, initial, mild symptoms might have been overlooked. Incubation period refers to the time-interval from the time of infection to the time of onset of symptoms. However, there are many cases in which exact time of infection is not clear. When multiple instances of contact with other patients were reported, maximum and minimum incubation periods have been determined, based on the time-interval between the initial and the final time point of contact. This epidemic in Korea are composed of several index patients who were infected in foreign countries such as China, first-generation patients who were estimated to be infected by index cases, and second-generation patients who were estimated to be infected by first-generation patients. Index case means initially detected patient in the first outbreak cluster. The first patient responsible for the outbreak may not be an index case. The source of infection to which index patients in Korea were exposed needs to be identified in further investigation and this study does not include it.

The generation time or serial interval means the time-interval between the date of symptom-onset of an index case and the date of symptom-onset of the subsequently infected patient.

The terms of “quarantine” and “isolation” needs to be carefully distinguished. In this report, the term “quarantine” is used to indicate that cases were selected as control target and segregated by the quarantine authority as contacted persons, considering their history of contact with another confirmed patient and/or their visit to the area of the outbreak (Wuhan city or the Hubei province). “Isolation” is used when the cases were confirmed to have been infected and segregated in a medical institute as patients.

### Ethics statement

The ethical approval or individual consent was not applicable. Data published by the KCDC were used in this study, and therefore consents from individual patients have not been obtained.

## RESULTS

### Epidemiologic characteristics

**Figure 1.**
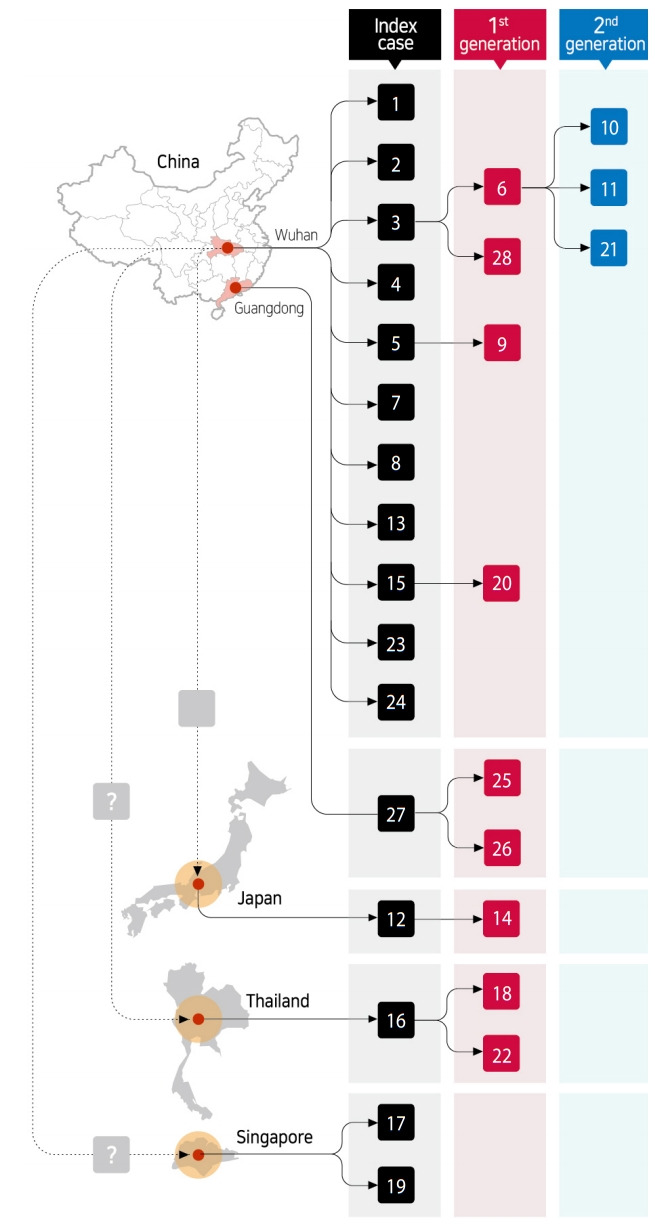
Early 28 cases of 2019 novel coronavirus disease in Korea according to the chain of transmission.

Examination of demographic characteristics of the 28 confirmed patients in Korea showed that 15 (53.6%) and 13 (46.4%) were male and female, respectively. Six of the patients were Chinese nationals, with three identified as visitors from China, while the other three were residents in Korea. The remaining 22 patients (78.6%) were Korean nationals. The median age of all patients was 42 years (21-73) (all adults, with 6, 6, 6, 8, 1 and 1 patient in their 20s, 30s, 40s, 50s, 60s and 70s, respectively) (Table 1). In total, 16 patients constituted index cases, while 9 and 3 were first-generation and second-generation patients, respectively (Figure 1).

Geographical regions in which 11 (68.8%), 1, 1, 1, and 2 index cases are speculated to have been infected include Wuhan, Guangdong, Japan, Thailand, and Singapore, respectively. Of the twelve first-generation and second-generation patients, 8 (66.7%) were family members of the index cases, while the remaining 4 patients were acquaintances who had been in close contact with index patients. All of these patients were identified during a control process for close contacts after confirmation index cases (Figure 1).

**Figure 2.**
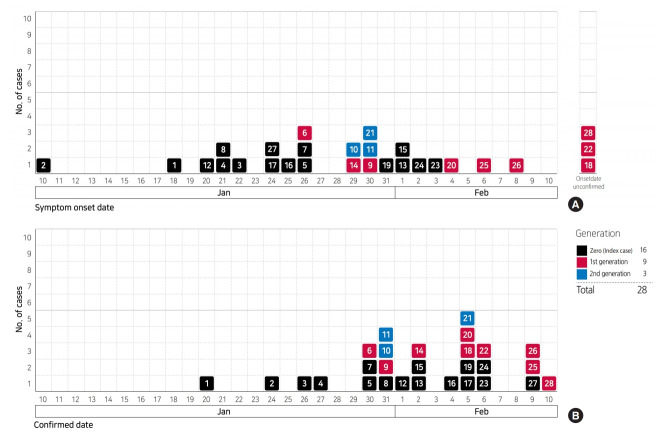
Epidemic curves of 2019 novel coronavirus disease outbreak for early 28 cases, Korea. (A) Curve according to symptom-onset date, (B) curve according to disease-confirmed date. The symptom-onset of #2 case was on January 10, 2020 in China and he entered Korea on January 22, 2020.

**Figure 3.**
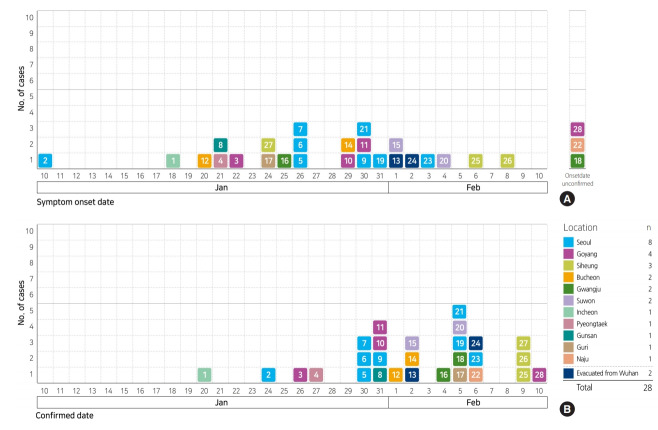
Epidemic curves of 2019 novel coronavirus disease outbreak for early 28 cases by area, Korea. (A) Curve according to symptom-onset date, (B) curve according to confirmed date. The symptom-onset of #2 case was on January 10, 2020 in China and entered Korea on January 22, 2020.

The epidemic curve of the outbreak according to date of symptom-onset ranges from January 10, 2020 to February 8, 2020, with January 26, 2020 showing the highest number of cases (n=3). However, dates of symptom-onset show a wide distribution, overall. The epidemic curve plotted according to the date of diagnosis, ranges from January 20, 2020 to February 10, 2020 and shows that the highest number of cases were on February 5, 2020 (Figure 2). On the other hand, patient #2 first developed symptoms on January 10, 2020 while in China, and was diagnosed on January 24, 2020, after his arrival in Korea on January 22, 2020. Thus, considering only those patients who developed onset of symptoms in Korea, the initial symptom-onset of the infection occurred on January 18, 2020, in patient #1. With regard to affected geographical regions, symptom-onset occurred in Incheon, Bucheon, Pyeongtaek, Gwangju, and Goyang in order (Figure 3).

**Figure 4.**
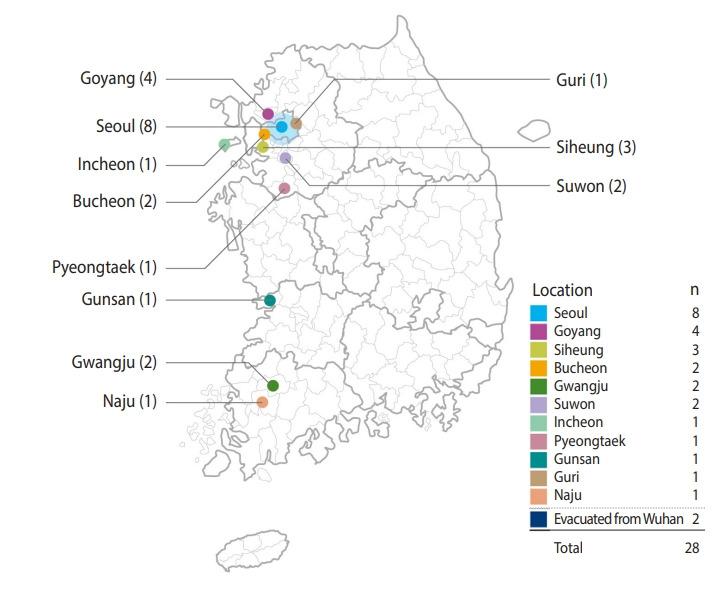
Early 28 cases of 2019 novel coronavirus disease by geographical area in Korea. The two cases that were evacuated from Wuhan were not included in this map.

Major areas where the diagnosed patients were exposed before being isolated, were Seoul and Gyeonggi-do with 8 and 13 cases (4, Goyang; 3, Siheung; 2, Bucheon; 1, Guri; 2, Suwon; 1, Pyeongtaek), respectively. The rest included Incheon, Gunsan, Gwangju, and Naju with 1, 1, 2, and 1 case, respectively (Figure 4). After identifying a tourist from Wuhan who visited Jeju-do (on January 21-25, 2020) and was confirmed of the infection on January 30, 2020 in China, 11 contacts of the patient were quarantined for 14 days, but were all released on February 8, 2020, after none were found to be infected.

**Figure 5.**
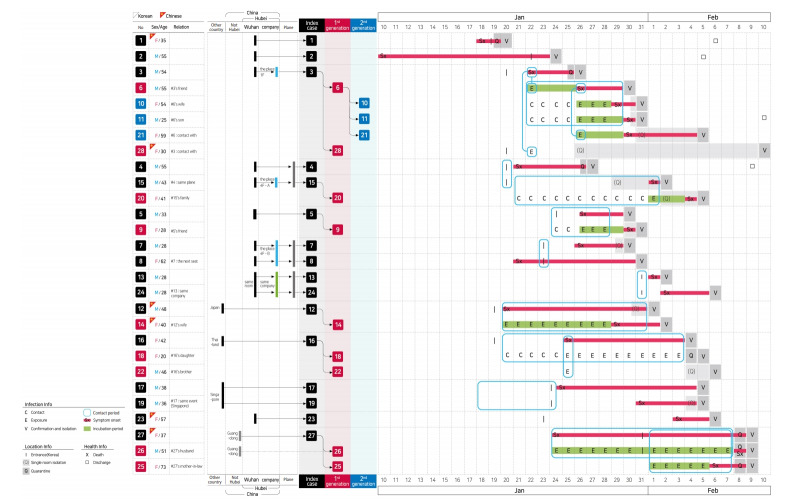
Summary of epidemiologic characteristics of early 28 patients diagnosed with 2019 novel coronavirus disease in Korea. M, male; F, female; Info, information.

The time-interval for entry into Korea for the 16 index cases ranged from January 19-31, 2020, excluding that for the 2 Koreans, who were transported from Hubei in a chartered aircraft, by the Korean government, along with other Korean citizens who had been residing there (Figure 5).

Incubation period is the time-interval between time of infection and onset of symptoms. However, as the exact time of exposure could not be ascertained in index patients and the time of symptom-onset could not be determined in those who were asymptomatic, these cases were excluded from the calculation. The estimates of incubation period included 0-15 days based on seven of the first-generation patients (#6, #20, #9, #14, #25, #26, and #28), and 1-4 days based on three of the second-generation patients (#10, #11, and #21), with mean and median values of 3.9 days (range 0-15) and 3.0 days, respectively (Table 1).

The mean and median serial interval was estimated to be 6.6 days (range 3-15) and 4.0 days, respectively, based on data of both first-generation and second-generation patients (Table 1).

The mean duration between symptom-onset and quarantine/isolation was 4.3 days (0-15). It was especially longer (10-12 days) in index patients who were infected in Japan, Thailand, and Singapore, which were not considered as possible risk-areas (Table 1).

Reproduction number (R) was estimated based on data of 26 (14, index; 9, 1st generation, and 3, 2nd generation) patients among total 28 confirmed cases, excluding the 2 Korean patients who had been immediately quarantined after being transported from Wuhan and therefore did not come into contact with the Korean population. As of now, the R is estimated to be 0.48 in Korea (Poisson 95% confidence interval, 0.25 to 0.84) (Table 1).

As of February 8, 2020, patient #6 had transmitted the infection to the highest number (n=3; 2 family members and 1 acquaintance). As super spreading event is defined when a patient transmits the infection to 5 or more people, this case has not yet been observed in Korea (Figure 5).

Of the 28 infected patients diagnosed in Korea, 3 were asymptomatic. Of them, patients #18 and #22 were first-generation patients infected by patient #16 and these patients were asymptomatic even though they were tested positive with the virus at the beginning of quarantine.

Of 28 patients, 4 have been discharged as of February 10, 2020. Patients #2, #1, #11, and #4 were discharged on day-13 (February 5), 19 (February 6), 11(February 10), and 15 (February 9) of hospitalization, respectively (Figure 5). There are currently no clinically serious patients among those undergoing treatment. After more patients recover and are discharged in the future, further detailed epidemiologic information (such as mean duration of hospitalization, duration of disappearance of symptoms and viral shedding since hospitalization) would be suggested.

### Patient characteristics

Data on clinical progression, exposed contacts, and transmission of infection for each confirmed case are presented as Appendix 1.

Baseline characteristics are shown in Figure 5.

## DISCUSSION

Analysis of data on confirmed cases in Korea, has yielded an incubation period of 3.9 days and median patient age of 42-year, which are lower than the incubation period of 5.2 days and the median age of 59 years announced in China [5]. The older age of patients might have increased fatality rate in China. The novel Coronavirus outbreak which originated in China at the end of 2019, is now spreading around the world. Totally, 28 cases have been confirmed in Korea as of February 10, 2020. Of these, 12 were infected in China, while 4 contracted the infection in other countries. The remaining 12 patients were infected by these index cases and were diagnosed while being monitored by the quarantine authority as contacts of confirmed patients. Therefore, community transmission with an unknown infection-cycle has not yet been occurred in Korea. Furthermore, due to blockade of the Hubei province (enforced on January 23, 2020) and limitation of group tour imposed by the Chinese government (on January 27, 2020), there have been no confirmed cases among the people travelling to Korea from China, since January 24, 2020. However, early diagnosis of patient is urgent as index cases are entering Korea from countries other than China. Fortunately, the technique of molecular diagnosis of the virus using reverse transcriptase-polymerase chain reaction, was quickly developed in Korea and has been available in medical institutions including private institutes across the country since February 7, 2020. This will allow early identification of undetected patients, thus improving their prognosis as well as preventing community transmission. WHO announced a Public Health Emergency of International Concern worldwide on January 30, 2020 [6], with an aim to cope more effectively with infectious disease, through international collaboration. It is necessary for all the countries including China, to promptly share epidemiological information on this new disease. Our epidemiology research team will keep accumulating new data and updating related indices.

## Figures and Tables

**Table 1.** Summary of epidemiologic characteristics of 2019 novel coronavirus disease using early 28 cases in Korea

Characteristics	n (%)
Male	15 (53.6)
Age (yr)	
20-29	6 (21.4)
30-39	6 (21.4)
40-49	6 (21.4)
50-59	8 (28.6)
60-69	1 (3.6)
70-79	1 (3.6)
Nationality	
Korean living in Korea	22 (78.6)
Chinese living in Korea	3 (10.7)
Chinese travelers from Wuhan, China	3 (10.7)
Source of infection	
Index case (n=16)	
Wuhan, China	11 (68.8)
Guangdong, China	1 (6.3)
Singapore	2 (12.5)
Japan	1 (6.3)
Thailand	1 (6.3)
1st generation (n=9)	
#16	2 (22.2)
#3	2 (22.2)
#5	2 (22.2)
#15	1 (11.1)
#12	1 (11.1)
#15	1 (11.1)
2nd generation (n=3)	
#6	3 (100)

**Period category (d)**	**Average (range)/median**

Incubation period^1^	3.9 (0-15)/3.0
Serial interval	6.6 (3-15)/4.0
Symptom-onset to diagnosis^1^	5.2 (0-16)/4.0
Symptom-onset to quarantine or isolation^1^	4.3 (0-15)/3.0
Diagnosis to discharge^2^	13.0 (7-17)/12.5

**Reproduction number**	**Estimate (Poisson 95% CI)/[binominal 95% CI]**

Total	0.48 (0.25, 0.84)/[0.28, 0.69]
1st generation (n=9)	0.56 (0.26, 1.07)/[0.30, 0.80]
2nd generation (n=3)	0.33 (0.07, 0.97)/[0.07, 0.70]

CI; confidence interval.

1 Three asymptomatic cases were excluded.

2 First 8 discharge cases were included.

## SUPPLEMENTARY MATERIALS

Korean version is available at http://www.e-epih.org/.

## Appendix 1.

Characteristics of 2019-nCoV disease patients diagnosed in Korea
