# Supplementary material for: Epidemiologic characteristics of early cases with 2019 novel coronavirus (2019-nCoV) disease in Korea
Source: Epidemiol Health. 2020 Feb 9;42:e2020007. doi: 10.4178/epih.e2020007 (PMC7285424; doi:10.4178/epih.e2020007)
Supplement: Supplementary file 1 [file epih-42-e2020007-suppl.docx]

-Special Article-

**Epidemiologic characteristics of early cases with novel coronavirus (2019-nCoV) disease in Republic of Korea**

단축제목: **Epidemiologic characteristics of 2019-nCoV disease in Korea**

(한국의 신종 코로나 바이러스 초기환자의 역학적 특성)

Moran Ki; Task Force for 2019-nCoV

Department of Cancer Control and Population Health, Graduate School of Cancer Science and Policy, National Cancer Center, Goyang, Korea

한글 초록:

2020년 1월 20일 한국의 신종코로나바이러스 감염자가 확진된 이후 약 20일만에 28명이 확진되었다. 역학적 특성을 분석한 결과 남자가 15명(53.6%), 중앙연령이 42세(20-73세)이었다. 확진자중 지표환자가 16명(57.2%), 1세대환자 9명(32.1%), 2세대환자가 3명(10.7%)이다. 전파된 환자들은 모두 가족이거나 가까운 지인으로 밀접접촉을 하였던 사람들이었다. 지표환자 16명중 15명은 1월 19일-24일에, 1명은 1월 31일에 국내에 입국하였다. 환자들의 잠복기는 평균 3.9일 (중위값 3일), 감염재생산수(R)는 0.48로 추정되었다. 확진자 중 3명은 증상이 없는 상태에서 확진이 되었다. 향후 새로운 정보가 나오면 역학 지표들이 수정될 것이다. 새로운 감염병에 대한 효율적 대비, 대응을 위해서는 전세계 연구자들의 역학정보 공유가 필수적이다.

영문 초록:

In about 20 days since the diagnosis of the first case of the 2019 novel coronavirus (2019-nCoV) in Korea on January 20, 2020, 28 cases have been confirmed. Fifteen patients (53.6%) of them were male and median age of was 42 years (range, 20-73). Of the confirmed cases, 16, 9, and 3 were index (57.2%), first-generation (32.1%), and second-generation (10.7%) cases, respectively. All first-generation and second-generation patients were family members or intimate acquaintances of the index cases with close contacts. Fifteen among 16 index patients had entered Korea from January 19 to 24, 2020 while 1 patient had entered Korea on January 31, 2020. The average incubation period was 3.9 days (median, 3.0), and the reproduction number was estimated as 0.48. Three of the confirmed patients were asymptomatic when they were diagnosed. Epidemiological indicators will be revised with the availability of additional data in the future. Sharing epidemiological information among researchers worldwide is essential for

effcient preparation and response in tackling this new infectious disease.

서론

2019년의 마지막날 중국에서는 원인불명 폐렴 환자가 발생하였다고 공식적으로 발표하였다. 이 새로운 감염병의 최초 환자 발생이 언제 인지는 불분명하다. 중국 우한시의 의료진이 2019년 12월 1일부터 2020년 1월 2일까지 확인된 총 41명의 감염자에 대한 보고에 의하면 첫 감염자의 증상발생일은 12월 1일이지만 화난 수산시장 방문력은 없었다 [1]. 따라서 이 환자가 사람간 감염에 의한 것이라면 첫 환자는 이보다 먼저 있었을 것으로 추정된다.

이후 2020년 1월 7일 중국에서 이 폐렴 질환의 원인으로 새로운 코로나바이러스를 보고하였고 해당 바이러스의 정보를 전세계 연구자들에게 공개하였다 [2]. WHO는 이 새로운 바이러스의 이름을 잠정적으로 2019 novel coronavirus (2019-nCov)로 명명하였다. 1월 10일에 중국에서 이 새로운 감염병으로 인한 첫 사망자 보고가 있었다. 1월 13일에는 중국이외 국가로 처음 태국에서 환자보고가 있었는데 해당 환자는 우한의 수산시장 방문력이 없었다. 이후 1월 15일에 일본에서 환자 보고가 있었고 1월 20일에는 한국에서 첫 환자 보고가 있었다. 이후 2월 8일 현재까지 한국의 확진자는 총 24명으로 늘어났다 [3]. 이 새로운 감염병에 대한 역학적 특성은 알려진 바 없어 기존의 coronavirus 감염 중에 사스(Severe Acute Respiratory Syndrome)와 메르스(Middle East Respiratory Syndrome)와 비교해가며 특성을 파악해 나가고 있다. 새로운 감염병의 역학적 특성을 빠르게 파악하는 것은 질병의 확산을 막고 조기발견으로 환자들의 치료 결과를 좋게 할 수 있는 중요한 방법이다. 이를 위해서는 전세계의 역학자들이 서로가 가지고 있는 정보를 빠르게 공유하는 것이 필요하다. 이를 기반으로 이 새로운 감염병에 대한 다양한 역학적 특성을 찾고 이에 맞추어 감염병의 추가 확산을 막는 것이 가장 시급하고도 중요한 문제이다.

이 보고는 한국에서 1월 20일부터 2월 10일까지 발생한 28명의 환자 특성과 이를 기반으로 역학적 특성을 검토한 자료이다. 다만 향후 역학조사가 보완되면 일부 정보가 바뀔 수 있음을 유념해야 한다.

방법(Methods)

한국 질병관리본부에서 역학조사 결과를 보도한 내용과 언론에서 추가 확인된 자료 등 공개된 자료를 활용하였다 [3, 4]. 증상 발생 시점은 환자의 진술에 의한 것이므로 경미한 증상이 간과되었을 가능성도 있다. 잠복기(Incubation period)는 감염시점부터 증상발생시점까지를 의미한다. 하지만 감염시점을 명확히 하기 어려운 경우도 많다. 환자와 여러 번 접촉한 경우는 최초 접촉 시점과 마지막 접촉 시점을 기준으로 최대, 최소 잠복기를 제시하였다. 이번 유행은 한국에서는 중국 등 해외에서 감염되어 들어오는 여러 명의 지표환자(index case)와 이들에게 전염된 것으로 추정되는 1세대(1^st^ generation) 환자, 1세대 환자에게 감염된 것으로 추정되는 2세대 (2^nd^ generation) 환자들로 이루어져 있다. 지표환자는 유행 그룹(outbreak cluster)에서 첫번째 발견된 환자를 의미한다. 해당 유행의 원인이 된 첫번째 환자가 지표환자가 아닐 수도 있다. 우리나라 지표환자들이 어떤 감염원에게 노출되어 감염이 되었는지는 향후 밝혀져야 할 부분으로 본 연구에서는 포함하지 않았다.

세대기(generation time) 또는 연속감염기간 (serial interval)는 지표환자 증상발생일부터 다음 감염 환자의 증상발생일까지로 계산하였다.

접촉자격리(quarantine)와 환자격리(isolation)를 모두 단순하게 격리라고 표현하면 혼동이 올 수 있다. 이 논문에서는 환자와 접촉하였거나 오염지역(우한시 또는 후베이성) 방문자로 방역당국의 관리대상으로 선정되어 격리(quarantine)하는 경우는 접촉자격리, 확진되어 의료기관에 격리(isolation)하는 경우는 환자격리라는 용어를 사용하였다.

이 연구는 질병관리본부에서 공개한 자료를 사용한 논문으로 개별 환자에게 동의를 받지는 않았다.

결과(Results)

1. 역학적 특성

우리나라에서 확진된 환자들 28명의 인구학적 특성을 보면 남자가 15명(53.6%), 여자가 13명(46.4%)이었고, 중국에서 방문한 중국인 3명, 국내 거주 중국인 3명, 한국인 22명(78.6%)이었다. 연령분포는 중앙값이 42세이고 범위는 21세- 73세로 모두 성인이다(20대 6명, 30대 6명, 40대 6명, 50대 8명, 60대 1명, 70대 1명) (Table 1).

역학적 특성을 살펴보면 확진자 28명중 16명은 지표환자(index case)이고 1세대 환자가 9명, 2세대환자가 3명이다 (Fig.1).

지표환자들이 감염된 것으로 추정되는 지역은 중국 우한시가 11명(11/16=68.8%) 이고 중국 광둥성 1명, 일본이 1명, 태국이 1명, 싱가포르가 2명이다. 1세대와 2세대 감염자 12명중 8명(66.6%)이 지표환자의 가족이고 나머지 4명도 가까운 지인으로 밀접접촉(close contact)을 하였던 경우이다. 이들은 모두 지표환자 확진 후 접촉자 관리 과정에서 확인되었다 (Fig.1).

환자발생 역학 그래프를 보면 증상일 기준으로는 1월 10일부터 2월 8일까지이고 이중 26일과 30일이 3명으로 가장 높지만 전체적으로 환자들의 증상 발생이 넓게 퍼져 있는 양상이다. 진단일 기준으로 보았을 때는 1월 20일부터 2월 10일까지이고 이중 2월 5일에 가장 많았다 (Fig.2). 그런데 가장 먼저 1월 10일에 증상을 보였던 #2 환자는 중국에 있는 동안 증상이 나타났고 이후 1월 22일 한국에 입국하여 24일 진단을 받았던 경우이다. 따라서 한국내에서 환자들의 증상 시작만을 고려하면 첫 환자 증상 시작은 #1 환자의 1월 18일로 볼 수 있다. 지역별로는 인천, 부천, 평택, 광주, 고양 등의 순서로 환자들의 증상이 시작된 것을 알 수 있다 (Fig. 3).

확진자들이 격리되기전에 주요 노출된 지역은 경기도가 13명(고양시 4명, 시흥시 3명, 수원시 2명부천시 2명, 구리시 1명, 평택시 1명)으로 가장 많았고, 서울시 8명이었다. 그 외에는 인천시 1명, 군산시 1명, 광주시 2명, 나주시 1명 등이다 (Fig.4). 제주도에서는 중국 우한에서 제주관광을 왔던 (1월 21일-25일) 사람이 중국에 돌아가 확진(1월 30일)을 받았다는 연락을 받고 접촉자 11명을 14일간 접촉자격리하였으나 확진자없이 2월 8일에 격리 해제 하였다.

지표환자들의 국내 입국 시기는 1월 31일 한국 정부가 수송기를 이용하여 후베이성에서 데려온 교민 2명을 제외하면 14명이 모두 1월 19일부터 1월 31일까지이다 (Fig. 5).

환자들의 잠복기는 감염시점부터 증상발생시점인데 지표환자는 노출시점을 알 수 없고, 무증상자는 증상발생 시점을 알 수 없어 이들을 제외하고 계산하였다. 잠복기 추정치는 1세대 감염자 (#6, #20, #9, #14, #25, #26, #28) 7명에서 0-15일로 나타났고, 2세대 감염자 (#10, #11, #21) 3명에서 1-4일로 나타나, 평균 3.9일(범위 0-15일)이었고, 중위값은 3일이었다 (Table 1).

연속감염기간(serial interval)은 1세대, 2세대 감염자의 자료를 보았을 때 평균 6.6일(범위 3-15일), 중위값은 4일로 추정되었다 (Table 1).

환자들의 증상 발생후 격리(Quarantine or Isolation)까지의 기간은 평균 4.3일(0-15일)이었고, 특히 위험지역으로 생각하지 못했던 일본, 태국, 싱가포르에서 감염된 지표환자들의 증상 후 격리까지의 기간이 10일~12일로 길었다 (Table 1).

감염재생산수(R)는 전체 확진자 28명중 우한에서 수송한 교민 환자 2명은 국내에서 접촉자가 없으므로 제외하고 26명 (지표환자 14명, 1세대 감염자 9명, 2세대 감염자 3명)을 기준으로 추정하였다. 그 결과 현재까지 우리나라의 감염재생산수(R)는 0.48 (Poisson 95% 신뢰구간 0.25-0.84) 로 추정되었다 (Table 1).

2월 8일 현재까지 가장 많은 감염 전파를 한 환자는 #6환자로 가족2명과 지인 1명에게 전파하였다. 하지만 보통 슈퍼감염자(super spreading event)를 5명 이상 전파한 환자로 정의하므로 아직까지 한국에서 슈퍼감염자는 없다 (Fig.5).

현재까지의 환자 28명 중에 3명은 증상이 나타나지 않았다. 이중 #18, #22 환자는 #16의 1세대 감염자인데 격리 시작 시점에 바이러스는 확인되었으나 증상은 없었다.

28명의 환자중에 2월 10일까지 퇴원 환자는 4명이다. 이중 2번환자가 2월 5일 입원 13일째에, 1번환자는 2월 6일 입원 19일째에 퇴원하였다 (Fig.5). 현재 치료중인 환자중에 중증으로 위중한 환자는 없다. 향후 퇴원환자가 좀더 누적되면 입원후 퇴원까지의 평균기간, 입원후 증상소실, 입원후 바이러스 배출 소실 등까지의 기간 등 좀더 자세한 역학적 정보를 제출할 수 있을 것이다.

2) 환자별 특징

확진자별 임상경과와 접촉자, 감염 전파 등은 Supplementary 로 제시하였다.

기본 특성은 그림 5에서 제시하였다.

토론(Discussion)

한국의 확진자 자료에서는 잠복기 3.9일 환자의 중위 연령이 42세로 중국에서 발표한 잠복기 5.2일보다 짧고, 중위 연령 59세 보다 낮다 [5]. 중국의 환자 연령이 높은 것이 치명률을 높이는 요인이 되었을 가능성이 있다. 이제 2019년말부터 중국에서 시작된 신종코로나바이러스 감염증이 전세계로 확산되고 있다. 한국에서는 2월 10일까지 총 28명이 확진되었고 이들 중 중국에서 감염된 사람은 12명이고 중국외 국가에서 감염된 사람이 4명이다. 나머지 12명은 이들을 통해 감염된 환자들이다. 지표환자에게 감염된 환자 9명은 모두 방역당국에 의하여 접촉자로 관리 중에 진단을 받았다. 따라서 아직까지 한국에서 감염고리를 알지못하는 지역사회 확산이 발생하지는 않았다. 또한 중국에서 후베이성 봉쇄(1월 23일)와 중국전체 단체여행객을 제한 (1월 27일)하는 조치로 인해 국내에는 1월 24일 이후 입국자에 대해서는 확진자 보고가 없는 상황이다. 다만 해외에서 감염된 지표환자가 중국이외 국가에서도 유입되고 있어서 조기진단이 매우 시급한 실정이다. 다행히 한국에서는 RT-PCR을 사용한 분자진단법이 빠르게 개발되어 2월 7일부터는 전국의 민간의료기관에서도 사용 가능하게 되었다. 이를 통해서 혹시라도 진단되지 않았던 환자들을 조기발견하여 환자들의 예후를 좋게하고 지역사회 확산을 예방할 수 있어야 할 것이다. WHO는 1월 30일에 전세계에 공중보건비상사태(Public Health Emergency of International Concern, PHEIC)를 선포하였다 [6]. 이는 국제공조를 통하여 더 효과적인 감염병대응을 하기 위한 것이다. 이를 위해서는 중국을 포함하여 모든 나라들이 이 새로운 질병의 역학정보를 빠르게 공유하는 것이 필요하다. 우리 역학연구팀은 계속 새로운 정보를 축적하여 지표를 수정해 나갈 예정이다.

ACKNOWLEDGEMENTS

방역에 애쓰고 있는 질병관리본부와 보건소 직원들, 현장에서 역학조사를 수행하고 있는 모든 역학조사관들의 노고에 깊이 감사드립니다.

자료 정리를 해준 이은영 연구원과 유행 상황을 이해하기 쉽도록 그림을 만들어 주신 오유숙 단오 대표님께 감사드립니다.

This research was supported by Government-wide R&D Fund project for infectious disease research (GFID), Republic of Korea (grant No. HG18C0088).

CONFLICT OF INTEREST

The authors have no conflicts of interest to declare for this study.

ORCID

Moran Ki http://orcid.org/0000-0002-8892-7104

REFERENCES

1.Huang C, Wang Y, Li X, Ren L, Zhao J, Hu Y, et al. Clinical features of patients infected with 2019 novel coronavirus in Wuhan, China. Lancet 2020

2.Carlos WG, Dela Cruz CS, Cao B, Pasnick S, Jamil S. Novel Wuhan (2019-nCoV) Coronavirus. Am J Respir Crit Care Med 2020

3.Yoo J-H, Hong S-T. The Outbreak Cases with the Novel Coronavirus Suggest Upgraded Quarantine and Isolation in Korea. Journal of Korean Medical Science 2020;35

4.KoreaCDC. Current status of 2019-nCoV outbreak in Republic of Korea [cited 2020 Feb 7]. Available from: <http://ncov.mohw.go.kr/tcmBoardList.do?brdId=&brdGubun=&dataGubun=&ncvContSeq=&contSeq=&board_id=>.

5.Li Q, Guan X, Wu P, Wang X, Zhou L, Tong Y, et al. Early Transmission Dynamics in Wuhan, China, of Novel Coronavirus-Infected Pneumonia. N Engl J Med 2020

6.WHO. Statement on the second meeting of the International Health Regulations (2005) Emergency Committee regarding the outbreak of novel coronavirus (2019-nCoV). Available from: <https://www.who.int/news-room/detail/30-01-2020-statement-on-the-second-meeting-of-the-international-health-regulations-(2005)-emergency-committee-regarding-the-outbreak-of-novel-coronavirus-(2019-ncov>). Updated 2020. Accessed Feb. 7, 2020.

| Table 1. Summary of epidemiologic characteristics of novel coronavirus (2019-nCoV) disease using early 24 cases in South Korea | | | |
| --- | --- | --- | --- |
| Characteristics |  |  |  |
|  |  | *n* | *%* |
| Male |  | 15 | 53.6 |
| Age | 20-29 | 6 | 21.4 |
|  | 30-39 | 6 | 21.4 |
|  | 40-49 | 6 | 21.4 |
|  | 50-59 | 8 | 28.6 |
|  | 60-69 | 1 | 3.6 |
|  | 70-79 | 1 | 3.6 |
| Nationality | Korean living in Korea | 22 | 78.6 |
|  | Chinese living in Korea | 3 | 10.7 |
|  | Chinese travelers from Wuhan, China | 3 | 10.7 |
| Source of infection |  |  |  |
| index case (n=16) | Wuhan, China | 11 | 68.8 |
|  | Guangdong, China | 1 | 6.3 |
|  | Singapore | 2 | 12.5 |
|  | Japan | 1 | 6.3 |
|  | Thailand | 1 | 6.3 |
| 1st generation (n=9) | #16 | 2 | 22.2 |
|  | #3 | 2 | 22.2 |
|  | #5 | 2 | 22.2 |
|  | #15 | 1 | 11.1 |
|  | #12 | 1 | 11.1 |
|  | #15 | 1 | 11.1 |
| 2nd generation (n=3) | #6 | 3 | 100.0 |
|  |  | *average (range)* | *median* |
| Incubation period (day)* |  | 3.9 (0-15) | 3.0 |
| Serial interval (day) |  | 6.6 (3-15) | 4.0 |
| Symptom onset to diagnosis (day)* |  | 5.2 (0-16) | 4.0 |
| Symptom onset to quarantine or isolation (day)* |  | 4.3 (0-15) | 3.0 |
| Diagnosis to discharge† (day) |  | 13.0 (7-17) | 12.5 |
| Reproduction number (R) | estimate | Poisson 95% CI | Binomial 95% CI |
| total | 0.48 | 0.25-0.84 | 0.28-0.69 |
| 1st generation (n=9) | 0.56 | 0.26-1.07 | 0.30-0.80 |
| 2nd generation (n=3) | 0.33 | 0.07-0.97 | 0.07-0.70 |
| CI; confidence interval.  * Three asymptomatic cases were excluded. | |  |  |

† First 8 discharge cases were included.

Appendix 1. 한국 2019-nCoV환자별 특성

**Case #1. (지표환자, 중국)**

**(#1환자의 임상 경과)**

#1 환자는 35세 여자로 중국 국적의 후베이성 우한시 거주자이다. 한국 입국 전날인 1월 18일부터 발열, 오한, 근육통 등에 대해서 증상을 보였으며, 같은 날 중국 우한시 의료기관에서 진료를 받고 감기 처방을 받았다. 1월 19일 12시 15분 CZ6079편으로 우한시에서 인천공항으로 입국했다. 입국 과정 중 인천공항검역소에서 중국 우한시 입국자에 대한 게이트 검역을 하는 과정에서 발열 등 증상이 확인되어, 방역당국에서 #1 환자를 조사대상 유증상자로 분류하고 인천의료원(국가지정입원치료병원)으로 이송 격리했다. 격리 후 판-코로나바이러스 PCR검사와 유전자 염기서열분석을 실시했고, 1월 20일 오전 신종 코로나바이러스로 확진 되었다. #1 환자는 중국 내 신종 코로나바이러스 진원지로 알려진 우한시 화난 해산물시장과 그 외 우한시 전통시장에는 방문한 이력이 없었으며, 중국 내 신종 코로나바이러스 확진 환자나 야생동물과 접촉한 적이 없었다고 하였다. 2월 6일 발열 등 증상이 호전되었고 2회 이상 실행한 신종 코로나바이러스 검사 결과 음성으로 확인되어 입원 격리 후 18일만에 퇴원하였다.

**(#1 환자의 접촉자 및 이들에 대한 방역)**

#1 환자의 접촉자는 환자와 같은 항공편을 이용한 승객 중 환자 좌석 앞, 뒤 3열을 포함한 총 7열에 탑승한 승객 29명, 승무원 5명, 공항 관계자 10명, 추가로 확인된 접촉자 1명 총 45명이다. 이 중 35명은 해당 보건소를 통해 유선으로 연락하여 발열, 호흡기 증상 여부를 모니터링하였다.

**(#1. 환자에 의한 감염 전파)**

#1 환자와 접촉한 45명 중 현재까지(2월 7일) 증상 발생자는 없었다. 접촉 후 잠복기 14일이 지났기 때문에 이들에 대한 모니터링은 종료되었다. 또한 #1 환자는 입국 검역 단계에서 신종 코로나바이러스 유증상자로 확인되어 바로 격리되었기 때문에 #1 환자에 의한 지역사회 노출은 없었다.

**Case #2. (지표환자, 한국)**

**(#2환자의 임상 경과)**

#2 환자는 한국 국적으로 55세 남자이다. 환자는 중국 후베이성 우한시에서 근무를 하고 있었으며, 1월 10일 처음 목감기 증상을 호소했다. 이 후 몸살 등의 증상이 심해져 1월 19일 우한시 현지 의료기관을 방문했고, 방문 당시 체온은 정상이었다. 1월 22일 중국 우한시를 출발하고 상하이를 경유해서 당일 저녁 FM823편으로 김포공항으로 귀국했다. 입국 당시 검역 과정에서 발열감시카메라상 발열 증상이 확인되어 건강상태질문서 징구 및 검역조사를 실시한 결과 발열(37.5도)과 인후통이 있었으나 호흡기 증상은 없어 능동감시 대상자로 분류 되었다. 1월 23일 인후통이 심해져 관할 보건소 선별진료소에서 진료를 받았고, X-ray 검사상 기관지염 소견이 확인되어 중앙역학조사관이 조사대상 유증상자로 분류했다. 이 후 국립중앙의료원에 격리되어 신종 코로나바이러스 검사를 실시한 결과 1월 24일 오전 신종 코로나바이러스 환자로 확진 되었다. 이후 인후통, 기침 등 증상과 흉부 x-ray 결과가 호전되었으며, 신종 코로나바이러스 검사를 24시간 간격으로 3회 실시한 결과 모두 음성으로 확인되어 2월 5일 한국의 확진자 중에 가장 먼저 퇴원하였다. #2 환자는 중국 우한시에 머무는 동안 중국 내 신종 코로나바이러스 진원지로 알려진 화난 해산물시장에 방문한 적은 없지만, 중국인 현지 직장동료가 감기 증상이 있었다고 하였다.

**(#2 환자의 접촉자 및 이들에 대한 방역)**

#2 환자와 접촉한 사람은 총 75명으로 항공기내 #2 환자와 인접하게 있었던 승객 등 56명, 공항 직원 4명, 자택 이동시 택시기사 1명, 자택 아파트 엘리베이터 동승자 1명, 보건소 직원 5명, 가족 2명, 추가 확인된 접촉자는 6명 등이다. 접촉자들은 증상 유무와 관계없이 관할 보건소에서 14일간 능동감시를 진행하였다.

**(#2. 환자에 의한 감염 전파)**

#2 환자와 접촉한 75명 중 현재까지(2월 7일) 증상발생자는 없었고 잠복기가 지나 모니터링은 종료되었다. 입국시 능동 감시 대상자로 분류된 이후 서울 자택에서 자가격리를 실시했기 때문에 #2환자에 의한 지역사회 노출은 없었다.

**Case #3. (지표환자, 한국, #6에 전파)**

**(#3 환자의 임상 경과)**

#3 환자는 한국 국적 54세 남자이다. 해당 환자는 중국 우한시에서 거주하다가 1월 20일 일시 귀국하였고, 귀국 당시에는 무증상이었다. 입국 당시 별다른 증상이 발견되지 않아 조사대상 유증상자로 분류되지 않았다. 1월 22일 오후 1시부터 열감, 오한 등 몸살기를 느껴 해열제를 복용하면서 지내며 증상이 다소 조절되는 듯했으나 1월 25일 간헐적 기침과 가래증상이 발생하여 질병관리본부 콜센터 1339로 자진 신고하였다. 관할 보건소의 1차 조사 결과를 바탕으로 역학조사관이 조사대상 유증상자로 분류했고, 1월 25일 명지병원으로 격리되어 신종 코로나바이러스 검사를 실시하였고, 1월 26일 확진되었다. #3 환자는 우한시 거주 중 우한시 소재 의류상가(더 플레이스)에서 근무한 이력이 있으며, 현재 신종 코로나바이러스 확진 환자 중 본인을 포함한 #7, #15 환자가 더 플레이스에 근무했고, # 8 환자는 해당 상가를 종종 방문하였다고 한다. #3 환자는 2월 7일과 11일 두차례 걸친 검사결과 음성으로 판정되어, 확진 판정 이후 17일이 지난 2월 12일에 퇴원했다.

**(#3 환자의 접촉자 및 이들에 대한 방역)**

증상 발생일(1월 22일)부터 격리(1월 25일) 전까지 #3 환자와 접촉한 사람은 총 98명으로 서울 강남구 소재 글로비 성형외과 직원과 환자 58명, 서울 강남구 호텔뉴브에서 체류하면서 접촉한 호텔 직원과 투숙객 12명, 압구정로 한일관(음식점) 4명, 도산대로 본죽(음식점) 2명, GS25시 한강잠원점(편의점) 1명, 가족과 지인 3명, 그 외 18명 등이다. 이 중 호텔 직원 1명이 유증상자로 확인되어 국가지정입원치료병상으로 격리되었으나, 신종 코로나바이러스 검사 결과 음성으로 확인되어 격리해제 되었다. 나머지 접촉자는 자택에서 접촉자 격리 (가족, 동행한 지인 등 14명) 및 능동감시를 실시했다.

**(#3. 환자에 의한 감염 전파)**

#3 환자와 접촉한 98명 중 1월 22일 한일관에서 같이 점심식사를 한 지인이 #6 환자로 확진 되었다. 한국에서 처음 발생한 사람간 전파이다. 이후 #6 환자의 가족(부인과 아들)이 확진 되었다(#10, #11). 그 외 접촉자는 2월 8일까지 추가환자발생없이 모니터링이 종료되었다. 하지만 2월 10일에 #3환자와 강남에 있는 성형외과를 같이 방문했던 지인(#28)이 코로나19 확진 되었다

**Case #4. (지표환자, 한국)**

**(#4 환자의 임상 경과)**

#4 환자는 한국 국적 55세 남자이다. #4 환자는 중국 우한시 방문 후 1월 20일 16시 25분 KE882편으로 인천공항으로 귀국했고 당시에는 특별한 증상이 없었다. 1월 21일 콧물 그리고 몸살 증세로 경기도 평택시의 평택 365 연합의원에 내원하여 치료를 받았다. 1월 25일 고열(38도)와 근육통이 발생하여 해당 의원에 재 방문하여 우한 방문력을 밝히고 능동감시를 받게 되었다. 1월 26일 근육통 악화 등으로 보건소 선별진료소에서 폐렴을 진단받고 신종 코로나바이러스 조사대상 유증상자로 분류되었으며, 같은 날 분당서울대병원으로 격리된 뒤 검사를 시행하여 1월 27일 오전 확진되어 입원하였다. 2월 9일 오전 24시간 간격으로 시행한 두 차례의 검사에서 음성 판정을 받아 퇴원했다.

**(#4 환자의 접촉자 및 이들에 대한 방역)**

#4 환자와 접촉한 사람은 총 172명으로 95명은 밀접접촉자, 77명은 일상접촉자로 분류되었다. 접촉 장소는 #4 환자가 탑승한 우한발 직항편 비행기, 17시30분에 인천공항에서 평택으로 출발한 8834번 공항버스, 평택 송탄터미널, 택시, 평택 365 연합의원, 평택 소재 보건소 등이다. 총 접촉자 172명 중 가족 1명을 포함한 총 3명이 유증상자로 확인되어 격리조치 후 신종 코로나바이러스 검사를 실행하였으나, 모두 음성으로 확인되었다. 나머지 접촉자들은 자택에서 접촉자격리 상태로 모니터링을 실시했다.

**(#4. 환자에 의한 감염 전파)**

#4 환자와 접촉한 172명 중 현재까지(2월 8일) 2차 감염자는 발생하지 않았고 2월 9일 격리 해제 예정이다. 환자가 1월 21일 증상 발현으로 인해 평택 365 연합의원에 내원하였음에도 불구하고 바로 확진검사가 시행되지 못한 이유는 당시 진료 의사가 DUR을 통해 우한 방문력을 확인하고 질문하였으나 환자가 단순히 중국을 다녀왔다고 대답하여 당시 검사대상 기준(우한 등 후베이성 방문자는 발열, 호흡기 증상자에게 검사, 그 외 중국 방문자는 폐렴 의심시에 검사)에 맞지 않았기 때문이다.

**Case #5. (지표환자, 한국, #9에 전파)**

**(#5 환자의 임상 경과)**

#5 환자는 한국 국적 33세 남자이다. 우한시에 업무차 방문 후 1월 24일 오전 5시 우한시 인근 장사 공항에서 출발하여 아시아나 항공 OZ322편으로 인천공항으로 귀국했다. 평소에 천식이 있어 간헐적인 기침이 있었고 입국 당시 발열이 없었기 때문에 능동감시자로 분류되었다. 1월 26일 오후부터 몸살 기운이 나타났고, 1월 29일 서울 중랑구 보건소 선별진료소로 이동 후 신종 코로나바이러스 검사를 받은 후 자택으로 귀가했다. 1월 30일 확진 되어 서울의료원에 입원하였다.

**(#5 환자의 접촉자 및 이들에 대한 방역)**

#5 환자와 접촉한 사람은 총 35명이고 이들은 자가격리 및 모니터링을 실시하고 있다. 총 35명 중 2명이 유증상자로 확인되어 격리조치 후 신종 코로나바이러스 검사를 실행하였으나, 1명은 음성이었으나 1명은 환자로 신종 코로나바이러스를 확진 받았다(#9 환자). 그리고 #5 환자가 장시간 체류한 버스, 음식점, 슈퍼마켓 등에 대해서는 환경소독을 완료하였다.

**(#5 환자에 의한 감염 전파)**

#5 환자와 접촉한 35명 중 1명이 1월 31일 신종 코로나바이러스 #9 환자로 확진 되었다. 한국에서 두번째 확진된 사람간 전파 감염자이다.

**Case #6. (1세대 감염자, 한국, #3로부터 감염됨. #10, #11, #21에 전파)**

**(#6환자의 임상 경과)**

#6 환자는 한국 국적 55세 남자이며, 한국에서 처음 발생한 사람간 전파 감염자이다. #6 환자는 1월 22일 서울 강남구 한일관에서 #3 환자와 93분간 함께 식사를 했다. #6 환자의 정확한 증상 발생일은 질병관리본부에서 아직까지 발표하지 않았으나, 현재까지 보도된 역학조사 결과 #6 환자의 증상 발생일은 1월 26일로 추정된다. 1월 26일 #3 환자 확진 후 일상접촉자로 분류되어 능동감시 중이었으나, 역학조사 과정에서 #3 환자의 증상 발현 시간이 저녁 7시에서 오후 1시로 변경되면서 1월 27일 능동감시에서 자가격리로 변경하여 관할 보건소에서 관리했다. 이후 신종 코로나바이러스 검사를 실시하여 1월 30일 확진 되었다.

**(#6 환자의 접촉자 및 이들에 대한 방역)**

#6 환자의 접촉자는 총 25명이고, 이 중 가족 2명(아내와 아들)이 신종 코로나바이러스 검사 결과 1월 31일 #10, #11 환자로 확진 되어 서울대병원으로 격리되었으며, 나머지 접촉자들은 자가격리와 모니터링을 실시했다. 2월 6일에는 명륜교회에서 #6 환자와 접촉한 자가격리 대상자 중 1명이 #21 환자로 확진 되었다.

**(#6 환자에 의한 감염 전파)**

#6번 환자의 총 접촉자 25명 중 아내(#10)와 아들(#11)이 1월 31일 신종 코로나바이러스 환자로 확진 되었고, 2월 5일 교회지인인 #21 환자가 확진 되었다. #6 환자는 한국에서 첫번째 2세대 감염전파를 일으킨 사례이며 지금까지 가장 많은 3명에게 감염을 전파한 사례가 되었다.

**Case #7. (지표환자, 한국)(#7 환자의 임상 경과)**

#7 환자는 한국 국적으로 28세 남자이다. 중국 우한시에서 청도를 거쳐 1월 23일 22시 20분 칭다오 항공 QW9901편으로 인천공항으로 귀국했고, 당시에는 별다른 증상이 없었다. 1월 26일 기침 증상이 약간 있었으며, 1월 28일 감기 증상을 시작으로 1월 29일 발열(37.7도), 기침, 가래 등의 증상이 나타났다. 1월 29일 증상이 호전되지 않아 관할 보건소에 신고를 했고, 보건소 선별진료소에서 조사대상 유증상자로 분류, 자가격리와 신종 코로나바이러스 검사를 실시했다. 1월 30일 자택에서 자가격리 중 당일 저녁 신종 코로나바이러스 환자로 확진을 받고 서울의료원으로 격리되었다. 2월 15일 완치 판정을 받고 퇴원했다.

**(#7 환자의 접촉자 및 이들에 대한 방역)**

#7 환자와 접촉한 사람은 총 21명이고, 이 중 6명은 유증상자로 확인되어 신종 코로나바이라스 검사를 실시했으나 검사 결과 모두 음성으로 나타났다. 접촉자들은 자가격리 및 모니터링을 실시했다.

**(#7 환자에 의한 감염 전파)**

#7 환자는 증상이 발현된 1월 26일 이후에는 주로 자택에 머물렀기 때문에 지역사회 접촉자는 매우 적었고 아직까지 #7 환자에 의한 2차 감염은 없다.

**Case #8. (지표환자, 한국)**

**(#8 환자의 임상 경과)**

#8 환자는 한국 국적 62세 여자이다. 1월 21일 중국 우한시 체류 중 근육통 증상이 발생했다. 1월 23일 인천공항으로 귀국하였고, 1월 27일 발열, 기침 등 증상으로 군산 유남진내과를 내원해 진료를 받았다. 1월 28일 발열, 기침 등 증상이 지속되어 군산의료원을 내원해 진료를 받았다. 진료 결과 조사대상 유증상자로 분류되어 신종 코로나바이러스 검사를 받았지만 음성으로 확인되어 자택으로 귀가하였다. 이후 1월 30일 발열, 기침 등 증상이 호전되지 않아 익산 원광대병원을 내원했고, 다시 조사대상 유증상자로 분류되어 입원하였고 1월 31일 확진 되었다. 2월 12일 완치 판정을 받고 퇴원했다.

**(#8 환자의 접촉자 및 이들에 대한 방역)**

#8 환자의 접촉자는 총 113명이고, 접촉자 중 3명이 유증상자로 확인되어 신종 코로나바이러스 검사를 실시했으나 검사결과 모두 음성으로 나타났다. 나머지 접촉자에 대해서는 자가격리 및 모니터링을 실시하였다. #8 환자는 증상 발현 후 군산 소재 의료기관, 서울 서초 소재 음식점, 군산 일대 대중목욕탕, 군산 소재 대형마트 등을 방문했고, 이에 대한 환경소독을 실시했다.

**(#8 환자에 의한 감염 전파)**

#8 환자와 #7환자는 중국 우한시에서도 알고 지낸 사이였다고하며, 1월 23일 귀국 당시 같은 비행기 옆 좌석에 앉았던 것으로 확인되었다. #7, #8 환자는 우한시 더플레이스에서 둘 다 감염된 것으로 추정된다. #7, #8 환자는 더 플레이스 4층에서 함께 근무를 했고, #3 환자는 1층에서 근무했는데, 주로 4,5층 화장실을 이용했고, #15 환자도 더 플레이스에 근무한 것으로 조사되었지만 정확히 몇 층에서 근무했는지는 알려지지 않았다. 보건 당국은 우한시 영사관을 통해 더 플레이스에서 근무하거나 머물렀던 한국인 50명도 파악해 조사중이다. 이들이 근무한 더 플레이스는 코로나바이러스 발원지로 알려진 화난시장과 차로 약 10~20분 거리에 있는 것으로 알려졌다

**Case #9. (1세대 감염자, 한국, #5로부터 감염됨)**

**(#9 환자의 임상 경과)**

#9 환자는 한국 국적의 28세 여자이다. #5 환자의 지인으로 1월 30일 #5 환자의 접촉자로 통보받고 자택에서 접촉자 격리 중에 증상이 발현되어 신종 코로나바이러스 검사를 실시했다. 1월 31일 확진 되어 서울의료원으로 이송 격리되었다.

**(#9 환자의 접촉자 및 이들에 대한 방역)**

#9 환자와 접촉한 사람은 총 2명이다. 접촉자는 자가격리 및 모니터링을 실시하고 있다.

**(#9 환자에 의한 감염 전파)**

#9 환자는 증상 발현된 1월 30일부터 확진 받은 1월 31일까지 자택에서 접촉자 격리중이었기 때문에 접촉자도 매우 적고 현재까지 감염 전파 보고는 없다.

**Case #10, #11. (2세대 감염자, 한국, #6로부터 감염됨)**

(#10, #11 환자의 임상 경과)

#10 환자는 한국 국적 54세 여자로 #6 환자의 아내이고, #11 환자는 한국 국적 25세 남자로 #6환자의 아들이다. #10 환자는 1월 29일 두통 증상을 보였고, #11 환자는 1월 30일 근육통 증상을 보였다. 1월 30일 점심쯤 자차 이용하여 경기도 일산 소재 미용실 이용 후 자택으로 귀가 후 #6 환자의 접촉자로 통보받고 자택에서 접촉자 격리 중에 신종 코로나바이러스 검사를 실시했으며, 1월 31일 확진 되어 서울대병원으로 격리되었다. #10, #11 환자는 한국에서 처음 발생한 2세대 감염자이다. #11환자는 2월 10일에 완치 판정을 받고 퇴원했다.

**(#10, #11 환자의 접촉자 및 이들에 대한 방역)**

총 접촉자는 39명이고, 접촉 장소는 경기도 일산 미용실과 명륜교회이다. 접촉자들은 자택에서 접촉자격리 및 모니터링을 실시하고 있으며, 해당 접촉 장소에 대해서는 환경소독을 완료했다.

**(#10, #11 환자에 의한 감염 전파)**

현재까지 #10, #11 환자에 의한 감염보고는 없다.

**Case #12. (지표환자, 중국, 일본에서 감염됨. #14에 전파)**

**(#12 환자의 임상 경과)**

#12 환자는 한국에 거주하는 중국 국적의 49세 남자이다. #12 환자는 업무상(관광 가이드) 이유로 일본에 체류하다가 1월 19일 김포공항을 통해 입국했다. 1월 20일 증상이 발현되었고, 1월 25일 군포 더건강한내과, 1월 28일 부천시 부선속내과를 내원하여 진료를 받았다. 하지만 증상이 호전되지 않았으며, 1월 30일 부천 보건소 선별진료소에서 신종 코로나바이러스 검사를 실시했다. 이후 부천시 순천향대학교부속병원을 방문하고 당일 17시부터 자가격리를 실시했다. 2월 1일 자택에서 자가격리 중 신종 코로나바이러스 환자로 확진되어 분당서울대병원으로 이송 격리되었다. #12 환자는 일본에 체류하는 동안 일본의 확진자와 같이 중국인 관광객과 접촉한 경험이 있다고 하였다. 또한 일본의 확진자는 일본에 방문한 중국인 관광객을 안내하던 기사로 일본에서 1월 28일에 확진을 받은 후에 #12 환자에게 1월 30일 연락하여 검사를 받아보도록 권하였다고 하였다. 이에 질병관리본부 콜센터 1339로 연락하여 보건소 선별진료소로 가게 되었다. 일본은 일본의 확진자와 같이 노출된 중국 국적자가 있음을 알고 중국에만 연락하여 우리나라 방역당국은 연락 받지 못한 상태였다. 이 부분은 일본 보건당국을 통해 확인되었다. #12 환자는 중국을 제외한 타국에서 감염되어 유입된 첫 번째 확진자이다.

**(#12 환자의 접촉자 및 이들에 대한 방역)**

#12 환자와 접촉한 사람은 총 219명이다. 이 중 #12 환자의 부인(#14)이 신종 코로나바이러스 환자로 확진 되었다. 나머지 접촉자는 자가격리 및 모니터링을 실시하고 있다

**(#12 환자에 의한 감염 전파)**

#12 환자에 의해 1세대 감염자(#14)가 발생했다. 또한 #12 환자는 중국이 아닌 일본에서 국내로 입국했고, 입국당시 증상이 없어 공항 검역에서 스크리닝 되지 못하였다. 1월 20일 증상 발현일부터 확진된 2월 1일까지 서울, 부천, 강릉, 수원, 군포 등 여러 지역을 방문했고, 사람이 많이 모여있는 남대문 쇼핑가, 극장, KTX 등을 이용했기 때문에 지역사회 전파가 우려되는 상황이었다. 하지만 2월 7일 현재까지 #12 환자의 부인인 #14 환자를 제외하고 추가 감염전파는 보고되지 않았다.

**Case #13. (지표환자, 한국)**

**(#13 환자의 임상 경과)**

#13 환자는 한국 국적 28세 남자이다. 1월 31일 중국 우한시에서 1차로 입국한 입국 교민 368명 중 한명이다. #13 환자는 입국 당시에는 증상이 없어 격리시설인 아산 경찰인재개발원으로 이송되었다. 1월 31일 1차 입국교민 전수 진단 검사 과정에서 신종 코로나바이러스 환자로 확진 되었으며, 국립중앙의료원으로 이송 격리되었다.

**(#13 환자의 접촉자 및 이들에 대한 방역)**

중국 우한시 교민 임시항공편 입국자로 격리된 상태에서 확진되어 현재까지 접촉자는 없다.

**(#13 환자에 의한 감염 전파)**

현재까지 #13 환자에 의한 감염 전파는 없다.

**Case #14. (1세대 감염환자, 중국, #12으로 부터 감염됨)**

**(#14 환자의 임상 경과)**

#14 환자는 중국 국적의 40세 여자이다. #12 환자의 아내로 1세대 감염자이다. 1월 29일 증상이 발현했으며, 2월 2일 자가격리 중 신종 코로나바이러스 환자로 확진되어 분당서울대병원으로 격리되었다.

**(#14 환자의 접촉자 및 이들에 대한 방역)**

#12 환자와 이동 경로가 동일하며, 접촉자는 3명으로 자가격리 및 모니터링을 실시 중이다.

**(#14 환자에 의한 감염 전파)**

현재까지 #14 환자에 의한 감염전파는 없다.

**Case #15. (지표환자, 한국, #20에게 전파)**

**(#15 환자의 임상 경과)**

#15 환자는 한국 국적 43세 남자이다. 1월 20일 16시 25분 KE882편으로 중국 우한시에서 인천공항으로 귀국했다. 중국 우한시에서 입국했기 때문에 능동감시 대상자였다. 이후 #4 환자 확진 후에 #4 환자의 항공기 접촉자로 확인되어 1월 29일부터 자가격리 중이었다. 2월 1일부터 발열, 가래, 인후통을 호소해 보건소 선별진료소를 방문했으며, 이후 신종 코로나바이러스 검사를 실시했고 2월 2일 확진되어 국군수도병원으로 격리되었다. #15 환자는 중국 우한시 소재 더 플레이스 의류매장 4층에서 매장을 운영하고 있었으며, #3 환자와 #7 환자는 해당 상가에서 근무를 했으며, #8 환자는 해당 상가를 종종 방문한 적이 있다고 밝혀졌다. 따라서 해당 상가에서 신종코로나바이러스 환자에게 노출된 것으로 보인다. 또한 #15 환자는 #4 환자와 같은 비행기를 타고 귀국한 접촉자이다. #4 환자의 증상 발생일이 1월 21일로 #15 환자와 #4 환자가 인천공항으로 귀국한 1월 20일 다음날이어서 #4 환자에 의한 전파가능성도 배제할 수는 없다.

**(#15 환자의 접촉자 및 이들에 대한 방역)**

#15 환자와 접촉한 사람은 총 14명이고, 이들 모두 자가격리를 실시하고 있다.

**(#15 환자에 의한 감염 전파)**

2월 7일 현재까지 #15번에 의해 1명(#20)이 신종 코로나바이러스에 감염된 것으로 보고되었다.

**Case #16. (지표환자, 한국, #18과 #22에게 전파)**

**(#16 환자의 임상 경과)**

#16 환자는 한국 국적 42세 여자로 폐암 병력이 있으며, 폐절제술을 받은 경험이 있다. 1월 15일부터 가족 5명과 태국(방콕, 파타야)을 여행하고 1월 19일 입국했다. 1월 25일 저녁부터 오한 증상이 발생하였고 1월 27일 9시경 발열 증상으로 광주21세기병원에서 진료를 받았다. 해당 병원에서 무릎인대 봉합수술 후 입원 중인 딸과 함께 1인실 입원실에 머물다 18시에 전남대병원 응급실을 방문하여 진료를 받았고 진료 후 22시에 광주21세기병원으로 이동했다. 1월 28일부터 2월 2일까지 딸 간병과 본인의 폐렴 진료를 위해 광주21세기병원에 체류했다. 체류 기간동안 외출은 거의 못하고 병원 내에서 입원 병실과 외래를 오가며 본인의 폐렴 치료를 받았다. 처음에는 딸이 입원한 1인실에서 지내다가 이후 딸과 엄마가 함께 2인실에 입원했다. 2월 3일 광주21세기병원에서 진료 결과 #16 환자의 임상 소견이 악화되어 전남대병원 응급실 선별진료소로 이동 후 신종 코로나바이러스 배제를 위해 격리조치를 실시했고 이후 광주 보건환경연구원에 의뢰해 신종 코로나바이러스 검사를 실시했다. 2월 4일 오전 전남대병원 음압병상 격리 중에 신종 코로나바이러스 환자로 확진 되었다. #16 환자가 태국을 방문한 시기는 1월 15일부터 19일까지인데 태국은 1월 13일 중국이외 국가들 중 가장 처음 환자를 보고한 나라이다. 또한 우한시에서 가장 방문객이 많은 도시가 방콕이다 [7]. 따라서 #16환자가 방콕에서 신종 코로나바이러스에 감염된 우한 여행자를 접촉했을 가능성이 있으며 또한 방콕 공항 등에서 접촉했을 가능성도 있다.

**(#16 환자의 접촉자 및 이들에 대한 방역)**

#16 환자와 접촉한 사람은 총 362명이다. 이중 고위험군으로 분류돼 21세기병원 내 격리된 사람은 25명(환자 20명, 보호자 5명), 저위험군으로 분류돼 광주소방학교 생활관에 격리된 사람은 34명이다. 그외에는 자택에서 접촉자 격리를 시행하고 있다. #16 환자의 접촉자 중 검사를 의뢰해 완료된 것은 2월 7일까지 160건이다. 함께 거주하고 있는 가족 4명과 친지 4명, 21세기병원·전남대병원 접촉자 145명, 광주소방학교 격리자 재검 7명 등이다. 이중 18번 환자인 16번 환자의 딸과 22번 환자인 친오빠를 제외한 158건은 모두 음성으로 나타났다. 접촉자 수는 16번 환자의 증상이 나타나기 시작한 1월25일 이후부터 2월3일까지 병원 CCTV, 신용카드 사용내역, GPS 기록 조회, 환자 면담 등을 통해 지속적으로 조사 중이어서 앞으로도 변동될 수 있다.

**(#16 환자에 의한 감염 전파)**

2월 5일 #16 환자의 딸(#18)과 2월 6일 #16 환자의 오빠(#22)이 신종 코로나바이러스 환자로 확진 되었으며, 2세대 감염자로 분류되었다.

**Case #17. (지표환자, 한국)**

**(#17 환자의 임상 경과)**

#17 환자는 한국 국적이고 38세 남자이다. 컨퍼런스 참석 차 1월 18일부터 싱가포르에 있었으며 1월 24일 8시50분 KE646편을 이용하여 인천공항으로 귀국하였다. 귀국 후 가벼운 감기 증상을 보였고 증상 발현 이후 마스크를 지속적으로 착용했다. 싱가포르 컨퍼런스 참석자 중 말레이시아 국적 확진자가 있다는 연락을 받고 2월 4일 한양대학교 구리병원에 있는 선별진료실을 방문하여 신종 코로나바이러스 검사를 실시했고 자택 격리 중 2월 5일 확진 되어 명지병원으로 격리되었다. #17환자는 코로나19로 확진 판정 후 8일이 지난 2월 12일에 완치 판정을 받고 퇴원했다.

**(#17 환자의 접촉자 및 이들에 대한 방역)**

# 17 환자와 접촉한 총 접촉자는 290명이고 모두 자가격리를 실시했다. #17 환자와 접촉한 사람은 많지만 #17 환자가 증상 발현 이후 줄곧 마스크를 쓰고 이동을 했고 그 결과 주변 밀접 접촉자들은 모두 신종 코로나바이러스 검사에서 음성으로 나타났다.

**(#17 환자에 의한 감염 전파)**

현재까지 #17 환자에 의한 감염 전파 보고는 없다.

**Case #18. (1세대 감염환자, 한국, #16으로부터 감염됨)**

**(#18 환자의 임상 경과)**

#18 환자는 한국 국적 20세 여자로 #16 환자의 딸이다. 1월 27일 광주21세기병원에서 무릎인대 봉합수술 수술 후 입원을 한 상태였으며 #18 환자와 1월 27일부터 2월 2일까지 증상이 발현된 #16 환자와 광주21세기병원 1인실과 2일실에 함께 머물렀다. 2월 4일 #16 환자가 신종 코로나바이러스감염증으로 확진된 후에 밀접접촉자로 격리되어 검사를 실시한 결과 2월 5일 확진 되었다. 확진 당시까지 특별한 증상은 보이지 않았다.

**(#18 환자의 접촉자 및 이들에 대한 방역)**

#18 환자의 접촉자는 총 4명이며 이들은 모두 접촉자격리를 시행 중이다. #18 환자는 병원에 입원중이었고 #16 환자 확진 이후 바로 격리되었기 때문에 주로 가족과 의료진만 접촉하였다.

**(#18 환자에 의한 감염 전파)**

2월 7일 현재까지 #18 환자에 의한 감염 전파는 보고되지 않았다.

**Case #19. (지표환자, 한국)**

**(#19 환자의 임상 경과)**

#19 환자는 한국 국적 36세 남자이다. #17 환자와 동일한 컨퍼런스 참석 차 1월 18일부터 싱가포르에 체류했고 1월 24일 #17 환자와 같은 비행기를 타고 인천공항으로 입국했다. 컨퍼런스에 참석했던 말레이시아 국적의 사람이 신종 코로나바이러스 환자로 확진 되었다는 통보를 받고 관할 보건소로 연락했다. 1월 31일부터 가벼운 증상이 발생하였으나 일상생활을 지속하였고 2월 4일부터 자가격리를 실시하다가 2월 5일 동일한 컨퍼런스에 참석한 #17 환자가 신종 코로나바이러스 환자로 확진 후 시행한 신종 코로나바이러스 검사에서 양성으로 확인되어, 서울의료원으로 격리되었다.

**(#19 환자의 접촉자 및 이들에 대한 방역)**

2월 7일 기준 #19 환자의 접촉자는 총 54명으로 모두 자택에서 접촉자 격리를 시행 중이다.

**(#19 환자에 의한 감염 전파)**

2월 7일까지 #19 환자에 의한 감염 전파 보고는 없다.

**Case #20. (1세대 감염자, 한국, #15으로부터 감염됨)**

**(#20 환자의 임상 경과)**

#20 환자는 한국 국적으로 41세 여자이며, #15 환자의 가족(처제)으로 2월 2일부터 자가격리 중 2월 5일 목 불편함 증상으로 보건소 선별진료소에 내원 후 시행한 신종 코로나바이러스 검사 결과 양성으로 확인되어 국군수도병원에 격리되었다. 2월 2일 #20 환자는 #15 환자와 접촉자로 분류되어 받은 1차 검사에서는 음성 판정을 받았지만 이 후 증상이 나타난 2월 5일 2차검사에서 양성으로 나왔다.

**(#20 환자의 접촉자 및 이들에 대한 방역)**

#20 환자와 밀접 접촉한 회사 동료 9명은 2월 2일부터 #15 환자의 접촉자로 재택근무 형식으로 자가격리를 실시하고 있어 #20환자로 인하여 추가된 접촉자는 없다.

**(#20 환자에 의한 감염 전파)**

2월 7일까지 #20 환자에 의한 감염 전파 보고는 없다.

**Case #21. (2세대 감염자, 한국, #6으로부터 감염)**

**(#21 환자의 임상 경과)**

#21 환자는 한국 국적 59세 여자이다. #6 환자와 1월 26일 명륜교회에서 접촉하였고, 1월 30일 증상이 발생하여 집에 머무르다 1월 31일 #6 환자의 접촉자로 확인되어 자택에서 접촉자 격리를 시작하였다. 접촉자 격리 중 2월 5일 인후통 증상으로 서울시 성북구 보건소 선별진료소에 내원하여 신종 코로나바이러스 검사를 실시하였고, 2월 5일 확진되어 서울대병원으로 이송 격리되었다

**(#21 환자의 접촉자 및 이들에 대한 방역)**

2월 7일 현재까지 접촉자 7명이 확인되어 자가격리 등 조치를 실시했다.

**(#21 환자에 의한 감염 전파)**

2월 7일 현재까지 #21 환자에 의한 추가 감염자는 보고되지 않았다.

**Case #22. (1세대 감염자, 한국, #16으로부터 감염)**

**(#22 환자의 임상 경과)**

#22 환자는 한국 국적 46세 남자이다. #16 환자의 가족(오빠)으로 2월 4일 #16 환자의 확진 후 자가격리 중 신종 코로나바이러스 검사를 실시했고 2월 6일 새벽 1시 검사결과 양성으로 확인되어 조선대병원에 격리되었다. #22 환자는 1월 25일 #16 환자, #18 환자를 포함한 가족 7명과 점심 식사를 했다. 2월 7일 현재까지 확인된 증상은 없어 무증상 감염으로 보인다. 2월 15일 완치 판정을 받고 퇴원했다.

**(#22 환자의 접촉자 및 이들에 대한 방역)**

2월 7일 현재까지 #22 환자의 접촉자는 1명(부인)으로 확인되었고, 부인의 신종 코로나바이러스 검사 결과는 음성이었다. #22 환자는 신종 코로나바이러스 확진을 받은 2월 6일 새벽 1시까지 직장과 대형마트를 방문했지만 #22 환자가 증상이 없어 직장동료 1명만 자가격리를 시행하였고, 대형마트에서 접촉한 사람에 대해서는 자가격리를 시행하지 않았다.

**(#22 환자에 의한 감염 전파)**

2월 7일 현재까지 #22 환자에 의한 감염 전파 보고는 없다.

**Case #23. (지표환자, 중국)**

**(#23 환자의 임상 경과)**

#23 환자는 중국 국적 57세 여자이다. 중국 우한시에서 1월 23일 단체 관광과 한국에서 유학중인 자녀를 만나러 입국했으며, 입국 당시에는 발열과 기침 등 호흡기 증상이 없었다. #23 환자와 동행자들은 우한시에서 온 입국자로 전수조사 대상자였으나 이들이 우한시가 봉쇄되어 돌아가지 못하고 한국 체류가 길어지면서 입국시에 제출한 기록에 있는 호텔에서 다른 곳으로 숙소를 이동하였기 때문에 방역당국이 소재를 파악하지 못하고 있었다. #23 환자는 2월 3일 증상이 발생했으며, 2월 4일 서울시가 경찰 협조를 받아 외국인 입국자에 대한 소재지를 파악하여 모니터링을 실시하는 과정에서 확인되었다. 이후 2월 5일 서대문구 보건소에서 신종 코로나바이러스 검사를 실시했고 2월 6일 양성으로 확진되어 국립중앙의료원으로 격리되었다.

**(#23 환자의 접촉자 및 이들에 대한 방역)**

#23 환자와 동행한 7명에 대해서 신종 코로나바이러스 검사를 실시했고 모두 음성이었다. 현재는 그들이 머물렀던 다가구주택에서 자가격리 조치 중이다.

**(#23 환자에 의한 감염 전파)**

2월 7일까지 #23환자에 의한 감염 전파 보고는 없다.

**Case #24. (지표환자, 한국)**

**(#24 환자의 임상 경과)**

#24 환자는 한국 국적 28세 남자이다. 1월 31일 중국 우한시에서 1차로 입국한 교민 368명 중 한명으로, 아산 경찰인재개발원에서 생활 중 이였으며, 입소 당시 시행한 신종 코로나바이러스 검사에서는 음성이었다. 시설에서 격리 생활 중 2월 6일 인후통 증상이 발생해 신종 코로나바이러스 검사를 실시했고 양성 판정을 받아 당일 저녁 9시 국립중앙의료원으로 격리되었다 #24 환자는 2월 2일 확진 판정을 받은 #13 환자와 중국 우한시에 함께 출장을 다녀온 직장 동료이고 출장 당시 한 숙소에서 2인 1실을 사용하며 생활 했다고 알려졌다. #13 환자와 1월 31일 1차 전세기편으로 귀국했고, 아산 경찰인재개발원 이동 당시 같은 버스를 타고 이동했다.

**(#24 환자의 접촉자 및 이들에 대한 방역)**

#24 환자는 시설에 입소한 이후에는 1인실에서 격리 생활을 하였기 때문에 접촉한 사람은 없다. 하지만 1월 31일 같은 버스로 이동한 교민들에 대해서는 2월 7일 현재 추가 검사가 시행되고 있다. 또한 이들과 같은 직장 동료 2명이 아산 시설에 있는 것으로 확인되어 추가 검사 중이며, 또한 중국에서 이들과 함께 일했던 다른 업체 직원들도 아산 시설에 다수 있는 것으로 확인되어, 이들에 대한 검사도 진행될 예정이다.

**(#24 환자에 의한 감염 전파)**

2월 7일 현재 역학조사가 진행 중이다.

**Case #25. (1세대 감염자, 한국 #27로부터 감염됨)**

**(#25 환자의 임상 경과)**

#25 환자는 한국 국적의 73세 여자이다. #25 환자는 2월 6일 발열, 기침, 인후통 등 증상이 발생했고 2월 7일 시흥시 신천연합병원 선별진료소를 내원했다. 이 당시에는 #25 환자는 중국 방문력이 없었고, 같이 사는 아들과 며느리가 중국을 방문을 했지만 우한시 방문자가 아니기 때문에 신종 코로나 바이러스 검사 대상자로 포함되지 않았다. 2월 8일 증상이 호전되지 않아 시흥시 신천연합병원을 다시 재방문 했으며, 코로나19 검사를 실시했다. 2월 9일 검사 결과 양성으로 확인되어 분당서울대병원으로 격리되었다. #25 환자는 중국 방문력은 없으나, 현재 함께 살고 있는 아들과 며느리가 중국 광둥성을 방문한 후 1월 31일 귀국했었다.

**(#25 환자의 접촉자 및 이들에 대한 방역)**

#25 환자는 증상 시작일 하루 전인 2월 5일부터 2월 9일 격리일까지 총 접촉자는 11명이다. 접촉자는 모두 자가격리를 실시했다.

**(#25 환자에 의한 감염 전파)**

현재 2월 11일까지 #25 환자에 의한 2차 감염자는 발생하지 않았다.

**Case #26. (1세대 감염자, 한국, #27로부터 감염됨), Case #27. (지표환자, 중국)**

**(#26. #27. 환자의 임상 경과)**

#26 환자는 한국 국적 51세 남자이며, #27 환자는 중국 국적 37살 여자이다. 이들은 부부이며, #25 환자와 한 공간에서 함께 거주했다. #26, #27 환자는 1월 31일 NX826편을 이용하여 마카오를 통해 인천공항으로 입국했고, 입국 당시에는 발열 증상은 없었다. #27 환자는 중국 체류 중인 1월 24일부터 기침 증상이 발생했고, 2월 5일 인후통, 기침과 발열증상으로 시흥시 신천연합병원 선별진료소를 방문하여 진료를 받았으나, 코로나19 검사가 아닌 인플루엔자 검사와 흉부방사선 촬영 검사를 받았고, 검사 결과 음성으로 나와 자택으로 돌아갔다. #27 환자는 2월 8일 인후통 증상이 발생했다. 2월 9일 한 공간에서 함께 거주하고 있는 #25 환자가 확진이 되었고, #26, #27 환자는 #25 환자의 밀접 접촉자로 분류 후 경기도의료원 안성병원으로 격리 되었고, 당일 코로나19 검사 결과 양성으로 확인 되었다.

#26, #27 환자는 무역업에 종사하며, 최근 후베이성 우한시를 방문한 적은 없고, 광둥성 체류 당시 병원, 시장을 방문한 적도 없었다. 또한 중국에서 체류하는 동안 야생동물을 섭취하지도 않았고 중국 사람은 만났지만, 신종 코로나 바이러스 확진 환자와는 접촉한 기억이 없다고 진술했다.

**(#26. #27. 환자의 접촉자 및 이들에 대한 방역)**

#26, #27 환자의 접촉자는 동일하며, 총 접촉자는 38명이다. 접촉자는 자가격리 및 모니터링을 실시했다.

**(#26. #27. 환자에 의한 감염 전파)**

#27 환자에 의해 #25 환자와 #26 환자가 감염된 것으로 보여 2세대 감염자로 분류되었다.

**Case #28. (1세대 감염자, 중국 #3로부터 감염됨)**

**(#28 환자의 임상 경과)**

#28 환자는 중국 국적 30살 여자이고, #3 환자와 함께 1월 20일 중국 우한에서 인천공항으로 입국했다. 입국 이후 #3 환자의 밀접 접촉자로 분류되어 1월 26일부터 자가격리 중이었다. 2월 8일 잠복기 완료 시점을 앞두고 코로나19 검사를 실시했고 검사 결과 양성과 음성의 경계선상의 결과가 나왔다. 2월 9일과 10일 2차례 재검사를 실시했으며, 2월 10일 최종적으로 양성으로 판정하고 명지병원에 격리되었다. #28 환자는 자가격리 중 발열이 확인되지 않았으며, 격리 전 #3 환자와 같이 방문한 성형외과에서 진통소염제를 일주일 처방받아 복용하고 있었기 때문에 증상 발현 시작일을 추정하기 어려운 상황이다.

**(#28 환자의 접촉자 및 이들에 대한 방역)**

#28 환자는 2월 10일 코로나19로 확진 받을 때까지 #3 환자 집에서 자가격리 중 이었고, 격리 중 함께 거주했던 접촉자 1명(#3 환자의 어머니)은 코로나19 검사결과 음성으로 나타났다.

**(#28 환자에 의한 감염 전파)**

현재까지 #28 환자에 의한 2차 감염자는 발생하지 않았다.

REFERENCES

1.Huang C, Wang Y, Li X, Ren L, Zhao J, Hu Y, et al. Clinical features of patients infected with 2019 novel coronavirus in Wuhan, China. Lancet 2020

2.Carlos WG, Dela Cruz CS, Cao B, Pasnick S, Jamil S. Novel Wuhan (2019-nCoV) Coronavirus. Am J Respir Crit Care Med 2020

3.Yoo J-H, Hong S-T. The Outbreak Cases with the Novel Coronavirus Suggest Upgraded Quarantine and Isolation in Korea. Journal of Korean Medical Science 2020;35

4.KoreaCDC. Current status of 2019-nCoV outbreak in Republic of Korea [cited 2020 Feb 7]. Available from: <http://ncov.mohw.go.kr/tcmBoardList.do?brdId=&brdGubun=&dataGubun=&ncvContSeq=&contSeq=&board_id=>.

5.Li Q, Guan X, Wu P, Wang X, Zhou L, Tong Y, et al. Early Transmission Dynamics in Wuhan, China, of Novel Coronavirus-Infected Pneumonia. N Engl J Med 2020

6.WHO. Statement on the second meeting of the International Health Regulations (2005) Emergency Committee regarding the outbreak of novel coronavirus (2019-nCoV). Available from: <https://www.who.int/news-room/detail/30-01-2020-statement-on-the-second-meeting-of-the-international-health-regulations-(2005)-emergency-committee-regarding-the-outbreak-of-novel-coronavirus-(2019-ncov>). Updated 2020. Accessed Feb. 7, 2020.

7.Wu JT, Leung K, Leung GM. Nowcasting and forecasting the potential domestic and international spread of the 2019-nCoV outbreak originating in Wuhan, China: a modelling study. Lancet 2020
