# Supplementary material for: Epidemiologic characteristics of early cases with 2019 novel coronavirus (2019-nCoV) disease in Korea
Source: Epidemiol Health. 2020 Feb 9;42:e2020007. doi: 10.4178/epih.e2020007 (PMC7285424; doi:10.4178/epih.e2020007)
Supplement: Supplementary file 2 [file epih-42-e2020007-app.pdf]

Appendix 1. Characteristics of 2019-nCoV disease patients diagnosed in Korea

Case #1. (Index case, China)

(Clinical progression of patient #1)

Patient #1 is a 35-year-old female of Chinese nationality, residing in Wuhan, in the Hubei province of China. She presented with symptoms of fever, chills, and muscle pain, which she was reportedly experiencing from January 18, 2020, since 1 day before her arrival in Korea. She had visited a medical center in Wuhan and had been advised medications for cold on the same day. On January 19, 2020. at 12:15 hours, she arrived at the Incheon Airport from Wuhan, by flight CZ6079. After disembarking, she was identified in the Incheon Airport National Quarantine Station during the gate quarantine process, implemented for checking those travelling to Korea from Wuhan. Considering that patient #1 was febrile, the quarantine authority classified her as a subject for investigation with presenting symptoms. She was transferred to the (Government-designated) Incheon Medical Center for isolation and treatment. After admission, polymerase chain reaction and further gene sequencing analysis for identification of pan-coronavirus, were conducted using the patient's blood samples, and her diagnosis was confirmed on the morning of January 20, 2020. Patient #1 did not report visiting the Huanan Seafood Wholesale Market (considered as the epicenter of 2019-nCoV disease outbreak in China) or any other traditional markets in Wuhan. She also reportedly had no exposure to wild animal or other confirmed patients of 2019-nCoV disease in China. As the patient's symptoms including fever, improved on February 6, 2020 and as the subsequently conducted two or more times of tests showed negative results, she was discharged after 18 days of hospitalization.

(Contacts of patient #1 and preventive measures for them)

Totally, 45 people had come into contact with patient #1. These included 29 passengers (who had travelled to Incheon on the same flight as the patient, while seated in her proximity including those in the same row and those seated in the consecutive three front and back rows), 5 flight-attendants, 10 airport-workers, and one additional contact. Of these, 35 people were monitored for occurrence of fever and respiratory symptoms by public health centers via landline calls.

(Transmission of infection by patient #1)

Of the people exposed to patient #1, none had become symptomatic as on February 7, 2020. The phone-based monitoring was terminated after completion of 14 days (incubation period) following the exposure. As patient #1 had been promptly identified and quarantined immediately on arrival in Incheon, she could not transmit the infection to the local population.

Case #2. (Index case, Korea)

(Clinical progression of patient #2)

Patient #2 is a 55-year-old male of Korean nationality. The patient was working in Wuhan, in the Hubei province of China and first reported experiencing sore throat on January 10, 2020. As his fatigue worsened, he visited a local medical center in Wuhan on January 19, 2020, though his temperature was found to be normal at the time. He left Wuhan on January 22, 2020 had a layover in Shanghai, and arrived at Kimpo Airport the same evening on flight FM823. During the quarantine processing upon arrival, the patient was found to be febrile as per the thermal screening system, and underwent a subsequent health-survey and physical examination. Although, he had fever (37.5°C) and sore throat, respiratory symptoms were absent, and the patient was classified as a subject for active surveillance. On January 23, 2020, the severity of sore throat increased, and he was treated at the screening clinic of a district health center. The patient's chest X-ray revealed signs of bronchitis, and central epidemiology investigator classified him as a subject for investigation of presenting symptoms. The patient was then isolated in the central medical center, tested for 2019-nCoV disease, and was confirmed to have the infection on the morning of January 24, 2020. Later, his symptoms including sore throat and cough resolved, and subsequent chest X-ray results showed improvement. Consequent blood tests for 2019-nCoV disease conducted twice, at 24-hourly intervals, were all negative. Therefore, the patient was discharged (first among all the confirmed patients in Korea) on February 5, 2020. Although patient #2 did not visit the Huanan Seafood Wholesale Market during his stay in Wuhan, he reported that his co-worker in China had been symptomatic with a cold.

(Contacts of patient #2 and preventive measures for them)

Totally, 75 people had come into contact with patient #2. These included 56 passengers (seated in proximity to the patient during flight), 4 airport-employees, 1 taxi-driver (who drove the patient to his house), 1 person (who shared an elevator with him at his apartment), 5 health-center employees, 2 family members, and 6 additional contacts. These contacts were actively surveilled by the district health center for 14 days, regardless of the presence of symptoms.

(Transmission of infection by patient #2)

Of the 75 people exposed to patient #2, none had developed symptoms as of February 7, 2020 and monitoring was terminated as the incubation period was over. As patient #2 had been classified as a subject for active surveillance upon arrival and quarantined in his home in Seoul, exposure to community did not occur.

Case #3. (Index case, Korea, infected patient #6)

(Clinical progression of patient #3)

Patient #3 is a 54-year-old male of Korean nationality. The patient was residing in Wuhan and had travelled to Korea on January 20, 2020, for a temporary visit. The patient was asymptomatic at the time of arrival and was not identified as a suspected case. As the patient started to feel fever, chills, and fatigue at 1:00 p.m. on January 22, 2020, he took an anti-pyretic, with which his symptoms were partially controlled. However, as the symptoms worsened to include an intermittent phlegmatic cough, the patient voluntarily contacted the call center (no. 1339) of KCDC on January 25, 2020, and notified his symptoms. Epidemiology investigator classified this patient as a subject for investigation with presenting symptoms, based on the results of his first-examination conducted at the district health center. On January 25, 2020, the patient was isolated in the Myongji Hospital and tested for 2019-nCoV disease. His diagnosis was confirmed on January 26. Patient #3 worked at a clothing store (The Place) located in Wuhan. Patients #7 and #15 also worked at The Place and patient #8 reported history of frequent visit to this store. Patient # 3 got the negative results from the two times of test on February 7, 2020 and 11, 2020 and was discharged on February 12, 2020 after 17 days of hospitalization.

(Contacts of patient #3 and preventive measures for them)

Totally, 98 people had come into contact with patient #3 between the date of symptom-onset (January 22, 2020) and the initiation of isolation (January 25, 2020). These included 58 employees and patients of the Glovi Plastic Surgery Clinic located in Gangnam (Seoul), 12 employees and customers of the Hotel Newv in Gangnam (Seoul), 4 people from the Hanilkwan restaurant in Apgujeong, 2 people from the Bonjuk restaurant in Dosandaero, 1 person from the GS25 convenience store at the Jamwon location along the Han River, 3 family members/acquaintances, and 18 other people. Of these, one of the hotel employees presented with symptoms and was isolated in a government-designated hospital for investigations and treatment. However, he was released after testing negative. Other contacts were quarantined in their homes (14 people including family members and acquaintances) and were actively surveilled.

(Transmission of infection by patient #3)

Of the 98 people who had come into contact with patient #3, the acquaintance who had lunch with him in the Hanilkwan restaurant on January 22, 2020 was later confirmed to be infected (patient #6). This was the first case of human-to-human transmission of 2019-nCoV disease in Korea. Subsequently, the wife (patient #10) and son (patient #11) of patient #6 were diagnosed with 2019-nCoV disease. Monitoring of remaining contacts was terminated on February 8, 2020 without occurrence of further cases. However, one acquaintance (patient #28), who visited plastic surgery clinic in Gangnam with patient #3, was confirmed with the infection on February 10, 2020.

Case #4. (Index case, Korea)

(Clinical progression of patient #4)

Patient #4 is a 55-year-old male of Korean nationality. Patient #4 arrived at the Incheon Airport on January 20, 2020, at 16:25 hours on flight KE882 after a visit to Wuhan and was asymptomatic at the time. On January 21, 2020, the patient visited the Pyeongtaek 365 Union Hospital in Pyeongtaek, Gyeonggi-do with symptoms of runny nose and fatigue and received treatment. On January 25, 2020, the patient visited the same hospital with high fever (38°C) and muscle pain. During this visit, he disclosed his complete travel history, and was identified as a candidate for active surveillance. On January 26, 2020 the patient was diagnosed with pneumonia at the screening clinic of public health center due to symptomatic aggravation with muscle pain and was classified as a subject for investigation with presenting symptoms. The patient was isolated in the Bundang Seoul National University Hospital on the same day, where he got test for 2019-nCoV disease. Then he was confirmed in the morning and hospitalized on January 27, 2020.

(Contacts of patient #4 and preventive measures for them)

A total of 172 people had come into contact with patient #4, and were grouped as close (n = 95) and daily (n = 77) contacts. The sites of contact included the direct flight that patient #4 took from Wuhan, the airport limousine (no. 8834) that left the Incheon airport at 17:30 hours. for the Songtan Terminal in Pyeongtaek, the taxi, the Pyeongtaek 365 Union Hospital, and a health-center located in Pyeongtaek. Of total 172 contacts, 3 including one family member presented with symptoms and were quarantined and tested. However, all three tested negative. Other contacts were quarantined in their homes and monitored.

(Transmission of infection by patient #4)

Of the 172 people who had come into contact with patient #4, no patient has been diagnosed as of February 8, 2020. They will be released from quarantine on February 9, 2020. The patient was not tested for 2019-nCoV disease right away during his first visit to the Pyeongtaek 365 Union Hospital on January 21, 2020 because, his symptoms did not meet the criteria for classifying him as a subject for investigation at the time (which included testing 1) travelers from the Hubei province/Wuhan, displaying fever and respiratory symptoms, and 2) travelers from China with suspected pneumonia). During the patient's first visit, he simply reported his visit to China to the examining doctor, who later confirmed his history of visit to Wuhan through drug utilization review.

Case #5. (Index case, Korea, infected patient #9)

(Clinical progression of patient #5)

Patient #5 is a 33-year-old male of Korean nationality. Following a business-visit to Wuhan, the patient boarded an Asiana Airlines flight no. OZ322 from the Changsha Airport near Wuhan on January 24, 2020 at 5:00 a.m. and arrived at the Incheon Airport. As the patient was afebrile but was found to have intermittent cough upon arrival (due to asthma), he was classified as a subject for active surveillance. He developed symptoms of fatigue in the afternoon of January 26, 2020, and visited the screening clinic at public health center in Jungnang-gu, Seoul on January 29, 2020. The patient went home that day, after being tested for 2019-nCoV disease. A positive result was obtained on January 30, 2020, and the patient was admitted to the Seoul Medical Center.

(Contacts of patient #5 and preventive measures for them)

Totally, 35 people come into contact with patient #5, and they were quarantined and monitored in their respective homes. Of these, 2 people presented with characteristic symptoms and were tested for 2019-nCoV disease after completion of quarantine. While one tested negative, the other was diagnosed with the infection (patient #9). Environmental disinfection was conducted on the buses, restaurants, and supermarkets where patient #5 reported spending longer periods of time.

(Transmission of infection by patient #5)

Of the 35 people who had come into contact with patient #5, one (patient #9) was diagnosed with 2019-nCoV disease on January 31, 2020. This was the second confirmed case of human-to-human transmission in Korea.

Case #6. (1st generation patient, Korea, infected by patient #3. Infected patients #10, #11, #21)

(Clinical progression of patient #6)

Patient #6 is a 55-year-old male of Korean nationality and was the first case of human-to-human transmission in Korea. Patient #6 had a meal with patient #3 in the Hanilkwan restaurant, located in Gangnam-gu, Seoul, for 93 minutes on January 22, 2020. While the exact date of symptom-onset in patient #6 has not been announced by the KCDC, epidemiologic study results that have been reported so far estimate it to be on January 26, 2020. Patient #6 had been under active surveillance as he had been classified as a daily-contact of patient #3 after the diagnosis of the latter with 2019-nCoV disease, on January 26, 2020. However, as the patient #3's time of appearance of symptoms was modified from 7:00 p.m. to 1:00 p.m. during epidemiologic investigation, patient #6's classification was changed from active surveillance to home quarantine on January 27, 2020, and the patient was monitored by the district public health center. He was later tested and confirmed on January 30, 2020.

(Contacts of patient #6 and preventive measures for them)

Totally, 25 people had come into contact with patient #6. Of these, his wife (patient #10) and son (patient #11) tested positive for 2019-nCoV disease on January 31, 2020, and were isolated in the Seoul National University Hospital. Other contacts were monitored under quarantine in their respective homes. On February 6, 2020, one of the home-quarantined subjects, who had been in contact with patient #6 at the Myeongnyun Church, was diagnosed with the infection (patient #21).

(Transmission of infection by patient #6)

Of the 25 contacts of patient #6, on January 31, his wife (patient #10) and son (patient #11), and on February 5, 2020 an acquaintance from his church (patient #21) tested positive. Patient #6 caused the first second-generation case of 2019-nCoV infection in Korea, who infected 3 people which is the highest number of transmission recorded till date.

Case #7. (Index case, Korea)

(Clinical progression of patient #7)

Patient #7 is a 28-year-old male of Korean nationality. He left Wuhan, had a layover in Chengdu, and arrived at the Incheon Airport on January 23, 2020 at 22:20 hours. on a Qingdao Airlines flight no. QW9901. He was asymptomatic at the time of arrival. The patient experienced a mild cough on January 26, 2020 and initial symptoms of cold on January 28, 2020. He developed fever (37.7°C) and a phlegmatic cough, on January 29, 2020. He reported to the district health-center on January 29, 2020 as his symptoms did not improve. The screening clinic of the district public health center classified the patient as a subject for investigation with presenting symptoms and quarantined him at home, after testing for 2019-nCoV disease. The patient was confirmed to be infected in the evening of January 30, 2020, and was isolated in the Seoul Medical Center. Patient #7 was discharged on February 15, 2020 after he was be completely cured.

(Contacts of patient #7 and preventive measures for them)

Totally, 21 people had come into contact with patient #7, and were monitored under home-quarantine. Of these, 6 developed characteristic symptoms and were tested for 2019-nCoV disease, and all results were negative.

(Transmission of infection by patient #7)

As patient #7 stayed mainly at home after January 26, 2020, since the appearance of his symptoms, contacts in community were very few, and no case of infection by patient #7 have been reported.

Case #8. (Index case, Korea)

(Clinical progression of patient #8)

Patient #8 is a 62-year-old female of Korean nationality. The patient developed muscle pain on January 21, 2020 during her stay at Wuhan. She arrived at the Incheon Airport on January 23, 2020 and visited the Yoo Nam-jin Hospital in Gunsan on January 27, 2020, for symptoms of fever and cough. On January 28, 2020, she visited Gunsan Medical Center for continuous symptoms such as fever and cough. examination, the patient was classified as a subject for investigation with presenting symptoms and was tested. However, the patient tested negative and was sent home. By January 30, 2020, her symptoms of fever and cough showed no improvement, and she visited the Wonkwang University Hospital for treatment. She was again classified as a subject for investigation with presenting symptoms, admitted, and re-tested, and was confirmed on January 31, 2020. Patient #8 was discharged on February 12, 2020 after he was be completely cured.

(Contacts of patient #8 and preventive measures for them)

Totally, 113 people came into contact with patient #8. While three of these, presented with symptoms, they tested negative. Remaining contacts were monitored under home-quarantine. Patient #8 visited a restaurant in Seocho (Seoul), as well as a medical center, a public sauna, and a mall in Gunsan, while she was symptomatic. Environmental disinfection was conducted at these sites.

(Transmission of infection by patient #8)

Patients #7 and #8 were acquaintances in Wuhan and were seated adjacent to each other in the same flight before their arrival in Korea, on January 23, 2020. Both these patients are suspected to be infected at a clothing store (The Place) in Wuhan, where they worked together on the 4th floor. Patient #3 also worked on the 1st floor of the same store and mainly used restrooms on the 4th and 5th floors. Patient #15 was also reported to have worked at The Place, although, the floor on which he worked is not known. The public health authority via the Korean Consulate in Wuhan, is also investigating 50 Koreans who reportedly worked or stayed at The Place. The store is located at a 10-20-minute drive from the Huanan Seafood Wholesale Market (epicenter of the outbreak).

Case #9. (1st generation patient, Korea, infected by patient #5)

(Clinical progression of patient #9)

Patient #9 is a 28-year-old female of Korean nationality and an acquaintance of patient #5. She was identified as a contact of patient #5, was notified, and quarantined at home from January 30, 2020. She subsequently developed symptoms and tested for 2019-nCoV disease. She was confirmed on January 31, 2020 and isolated in the Seoul Medical Center.

(Contacts of patient #9 and preventive measures for them)

Two people had come into contact with patient #9. They are currently under home quarantine and are being monitored.

(Transmission of infection by patient #9)

Patient #9 was already under home quarantine from January 30, 2020, when her symptoms appeared, until January 31, 2020, when she was confirmed with the infection. Therefore, contacts were few, and no case of transmission has been reported.

Cases #10 and #11. (2nd generation patients, Korea, infected by patient #6)

(Clinical progression of patients #10 and #11)

Patients #10 (54-year-old female) and #11 (25-year-old male) are both of Korean nationality and are the wife and son, respectively, of patient #6. Patient #10 developed a headache on January 29, 2020, while patient #11 experienced symptom of muscle pain on January 30, 2020. On January 30, 2020 they reportedly drove to a hair salon in Ilsan, Gyeonggi-do (around lunch-time) and back home. They were notified of their status as contacts of confirmed patient #6 and put under home quarantine, after the trip. They tested for 2019-nCoV disease and were confirmed on January 31, 2020 and were isolated in the Seoul National University Hospital. Patients #10 and #11 were the first cases of second-generation patients identified in Korea. Patient #11 was discharged on February 10, 2020 after he was be completely cured.

(Contacts of patients #10 and #11 and preventive measures for them)

Totally, 39 people came into contact with patients #10 and #11, at sites of a hair salon in Ilsan (Gyeonggi-do) and Myeongnyun Church. These contacts have been quarantined and are being monitored in their respective homes. Environmental disinfection was conducted at the sites of contact.

(Transmission of infection by patients #10 and #11)

There are currently no reported cases of infection transmitted by patients #10 and #11.

Case #12. (Index case, China, Infected in Japan. Infected patient #14)

(Clinical progression of patient #12)

Patient #12 is a 49-year-old male of Chinese nationality, residing in Korea. The patient was in Japan for business (tour-guide) and arrived at the Kimpo Airport on January 19, 2020. On developing symptoms on January 20, 2020, he visited the Duhgunganghan Hospital in Gunpo, on January 25, 2020, and the Bucheon Sok Hospital in Bucheon on January 28, 2020. However, the patient's symptoms showed no improvement, and he was tested for 2019-nCoV disease at the screening clinic of the Bucheon public health center on January 30. Later, the patient visited the Soonchunhyang University Hospital in Bucheon and was put under home-quarantine at 17:00 on the same day. The patient was confirmed on February 1, 2020, while under home-quarantine and was transferred to the Bundang Seoul National University Hospital for isolation. Patient #12 reportedly had come into contact with a Chinese tourist along with a confirmed Japanese patient during his stay in Japan. The confirmed Japanese patient was a driver guiding the Chinese tourist, who was visiting Japan. After being diagnosed with the infection in Japan on January 28, 2020, the Japanese patient contacted patient #12 on January 30, 2020, to suggest that he get tested for 2019-nCoV disease. Therefore, patient #12 contacted the call-center of the KCDC (no 1339) and went to a screening clinic of public health center. As the Japanese government had only informed Chinese officials after finding out that a Chinese citizen had been exposed in addition to the confirmed Japanese patient (driver), the Korean quarantine authority was not informed. This information was confirmed with the Japanese public health authority. Patient #12 is the first confirmed case infected in other country besides China.

(Contacts of patient #12 and preventive measures for them)

Totally, 219 people had come into contact with patient #12. Of these, his wife (patient #14) was diagnosed with 2019-nCoV disease. Other contacts are currently being monitored under quarantine in their respective homes.

(Transmission of infection by patient #12)

A first-generation patient #14 was infected by patient #12, in Korea. As patient #12 travelled from Japan (instead of from China) and was asymptomatic upon arrival, he was not screened during the airport quarantine process. The patient visited numerous places including Seoul, Bucheon, Gangneung, Suwon, and Gunpo from January 20, 2020 (when his symptoms first appeared) until February 1, 2020, when he was confirmed. As he visited heavily-crowded sites such as the Namdaemun shopping strip, a movie theater, and used KTX, spread of infection to local communities was a serious concern. However, as of February 7, 2020, with the exception of his wife (patient #14), no additional cases of infection have been reported.

Case #13. (Index case, Korea)

(Clinical progression of patient #13)

Patient #13 is a 28-year-old male of Korean nationality. He is one of the first round group of 368 Koreans who were brought back to Korea on charter flight by Korean government from Wuhan on January 31, 2020. Patient #13 was asymptomatic on arrival and was transferred to the Asan Police Human Resources Development Institute, which is a designated quarantine facility. During the process of complete examination of the first round group of Koreans who arrived from Wuhan, patient #13 was confirmed with 2019-nCoV disease and was transferred to the National Medical Center for isolation.

(Contacts of patient #13 and preventive measures for them)

As the patient was immediately quarantined after disembarking from a short flight that was chartered to transport Koreans back home from Wuhan, and was later diagnosed (in quarantine), no contact was existing so far.

(Transmission of infection by patient #13)

No infection by patient #13 occurred.

Case #14. (1st generation patient, China, infected by patient #12)

(Clinical progression of patient #14)

Patient #14 is a 40-year-old female of Chinese nationality, who is the wife of patient #12 and a first- generation patient. Her symptoms appeared on January 29, 2020, and was confirmed on February 2, 2020, while she was quarantined in her home. She was isolated in the Bundang Seoul National University Hospital.

(Contacts of patient #14 and preventive measures for them)

The route of travel of patient #14 was the same as that of patient #12. Three people who had come into contact with patient #14 are currently being monitored under home quarantine.

(Transmission of infection by patient #14)

Transmission by patient #14 has not been reported.

Case #15. (Index case, Korea, infected patient #20)

(Clinical progression of patient #15)

Patient #15 is a 43-year-old male of Korean nationality. He arrived at the Incheon Airport on flight KE882 from Wuhan, on January 20, 2020 at 16:25 hours. Considering that the patient had travelled from Wuhan, he was classified as a subject for active surveillance. After confirmation of patient #4, patient #15 was identified as an in-flight contact of the former and was quarantined at home from January 29, 2020. The patient developed fever, phlegm, and sore throat on February 1, 2020, and visited a screening clinic of public health center. He was later tested for 2019-nCoV disease and was confirmed as a positive case on February 2, 2020. He was thereafter transferred to the Korean Armed Forces Capital Hospital for isolation. Patient #15 was running a store on the 4th floor of The Place (clothing store) in Wuhan. Patients #3 and #7 also worked at this store while patient #8 was a frequent visitor to this store. The store was therefore considered to be the site of exposure to a case of 2019-nCoV disease, for the aforementioned group of patients.

In addition, patient #15 arrived in Korea on the same flight as patient #4. The date of symptom-onset for patient #4 was January 21, 2020, which is 1 day after patients #15 and #4 arrived at the Incheon Airport (January 20, 2020). Therefore, transmission by patient #4 could be a possibility.

(Contacts of patient #15 and preventive measures for them)

Totally, 14 people came into contact with patient #15, and they are currently being monitored under home quarantine.

(Transmission of infection by patient #15)

As of February 7, 2020, one person (patient #20) was reported to have been infected with 2019-nCoV disease, by patient #15.

Case #16. (Index case, Korea, infected patients #18 and #22)

(Clinical progression of patient #16)

Patient #16 is a 42-year-old female of Korean nationality, with a history of lung cancer and pneumectomy. The patient traveled to Thailand (Bangkok, Pattaya) on January 15, 2020, with 5 family members and arrived back in Korea on January 19, 2020. She developed chills on the evening of January 25, 2020, and visited the 21st Century Hospital in Gwangju, with the symptom of fever, on January 27, 2020, at around 9:00 hours. The patient stayed in a single-bed hospital room with her daughter, who was hospitalized for knee-joint syndesmorrhaphy and visited the emergency room of the Chonnam National University Hospital at 18:00. After treatment, the patient returned to the 21st Century Hospital in Gwangju at 22:00. The patient stayed in this hospital between January 28, 2020 and February 2, 2020 to care for her daughter and for her own pneumonia treatment. The patient could barely go out during this period and went back and forth between her daughter's hospital room and the outpatient clinic for her pneumonia treatment. While the patient initially stayed in the single-bed hospital room occupied by her daughter, both later moved to occupy a double-bed hospital room together. As the condition of patient #16 worsened with clinical progression, during her admission in the 21st Century Hospital, the patient was transferred to the emergency room/screening clinic of the Chonnam National University Hospital and was isolated, considering the risk of transmission. Later, the patient was tested by the Gwangju Health Environment Research Institute for 2019-nCoV disease. On February 4, 2020, patient #16 was confirmed during admission under isolation in the Chonnam National University Hospital. Patient #16 had visited Thailand between January 15, and 19, 2020, and Thailand was the first country after China, to report a 2019-nCoV disease case on January 13, 2020. Moreover, Bangkok is known to attract most tourists travelling from Wuhan [A1]. Therefore, patient #16 might have contact an already infected tourist from Wuhan in Bangkok or Bangkok airport.

(Contacts of patient #16 and preventive measures for them)

Totally, 362 people came into contact with patient #16. While 25 of these were classified as a high-risk group (20 patients, 5 guardians) and were isolated in the 21st Century Hospital, 34 were classified as a low-risk group and were quarantined at the civic center of the Gwangju Fire Service Academy. The remaining suspected contacts are being monitored under home quarantine. Of all contacts of patient #16, 160 have been tested as of February 7, 2020. They were her 4 family members who live together, 4 relatives, 145 contacts at the 21st Century Hospital and the Chonnam National University Hospital, and retest of 7 people already quarantined at the Gwangju Fire Service Academy. With the exception of her daughter (patient #18) and an older brother (patient #22), all remaining 158 patients tested negative for 2019-nCoV disease. The number of suspected contacts of patient #16 between January 25, 2020 (date of symptom-onset) and February 3, 2020, is still under investigation through examination of closed circuit television footage of both hospitals, tracking of credit card usage, study of global positioning system records, and patient-interviews and is therefore subject to change.

(Transmission of infection by patient #16)

On February 5, 2020, a daughter (patient #18) and an older brother (patient #22) of patient #16 were diagnosed with 2019-nCoV disease and classified as first-generation patients.

Case #17. (Index case, Korea)

(Clinical progression of patient #17)

Patient #17 is a 38-year-old male of Korean nationality. The patient stayed at Singapore from January 18, 2020, to attend a conference and arrived at the Incheon Airport on January 24, at 8:50 hours, on flight KE646. Following his arrival, the patient developed a mild cold and therefore consistently wore a mask after symptom-onset. After being informed that there was a confirmed case of a Malaysian national with 2019-nCoV disease among the conference attendees in Singapore, the patient visited a screening clinic at the Hangyang University Guri Hospital on February 4, 2020, and was tested for the virus. On February 5, 2020, the patient was confirmed to have the infection, while under home quarantine and was transferred to the Myeongji Hospital for isolation. The patient (#17) was discharged after 8 days of hospitalization on February 12, 2020. Up to now, he has been discharged the fastest among the infected patient in Korea.

(Contacts of patient #17 and preventive measures for them)

Totally, 290 people had come into contact with patient #17, and all of them quarantined in their homes. Despite the large number of contacts, patient #17 consistently wore a mask after symptom-onset and all his close contacts tested negative.

(Transmission of infection by patient #17)

Transmission of infection by patient #17 has not been reported.

Case #18. (1st generation patient, Korea, infected by patient #16)

(Clinical progression of patient #18)

Patient #18 is a 20-year-old female of Korean nationality, and a daughter of patient #16. Patient #18 was hospitalized at the Gwangju 21st Century Hospital, after undergoing knee-joint syndesmorrhaphy on January 27, 2020. The patient consecutively occupied a single-bed and double-bed hospital rooms with patient #16 (her mother) who was symptomatic between January 27, 2020 and February 2, 2020. After patient #16 was confirmed with 2019-nCoV infection on February 4, 2020 patient #18 was quarantined as a close-contact. The patient tested positive for the virus on February 5, although, she was asymptomatic at the time.

(Contacts of patient #18 and preventive measures for them)

Totally, 4 people had come into contact with patient #18, and all of them are under quarantine. As patient #18 was hospitalized at the time of exposure and was immediately isolated after her mother's diagnosis, her contacts consisted mainly of family-members and medical staff.

(Transmission of infection by patient #18)

As of February 7, infection by patient #18 has not been reported.

Case #19. (Index case, Korea)

(Clinical progression of patient #19)

Patient #19 is a 36-year-old male of Korean nationality. Patient #19 stayed at Singapore from January 18, 2020 to attend the same conference that was attended by patient #17, and arrived at the Incheon Airport on the same flight as that taken by the latter, on January 24, 2020. After being informed that there was a confirmed case of a Malaysian national with 2019-nCoV disease among the conference attendees in Singapore, patient #19 contacted his district public health center. Although he had developed mild symptoms on January 31, 2020, the patient continued with his daily activities and quarantined himself at home from February 4, 2020. Following diagnosis of patient #17 (who attended the same conference) with 2019-nCoV disease on February 5, 2020, patient #19 was also tested and found positive. He was moved to the Seoul Medical Center for isolation.

(Contacts of patient #19 and preventive measures for them)

Totally, 54 people had come into contact with patient #19 as of February 7, 2020, and all of them are currently being monitored under home quarantine.

(Transmission of infection by patient #19)

As of February 7, 2020, infection by patient #19 has not been reported.

Case #20. (1st generation patient, Korea, infected by patient #15)

(Clinical progression of patient #20)

Patient #20 is a 41-year-old female of Korean nationality, and is a family member (sister-in-law) of patient #15. During her home-quarantine period which began on February 2, 2020, the patient experienced symptomatic throat discomfort and visited a screening clinic of public health center (on February 5), where she tested positive for 2019-nCoV disease and was isolated in the Korean Armed Forces Capital Hospital for treatment. Patient #20 had tested negative on the first test performed on February 2, 2020 which was administered after her classification as a contact of patient #15. However, she tested positive on the second test performed on February 5, 2020, after symptom-onset.

(Contacts of patient #20 and preventive measures for them)

Totally, 9 co-workers who had been in close contact with patient #20 have already been home-quarantined as contacts of patient #15 since February 2, 2020 and have been teleworking. There was no additional contacts of patient #20.

(Transmission of infection by patient #20)

As of February 7, 2020, infection by patient #20 has not been reported.

Case #21. (2nd generation patient, Korea, infected by patient #6)

(Clinical progression of patient #21)

Patient #21 is a 59-year-old female of Korean nationality. The patient had come into contact with patient #6 on January 26, 2020 at the Myeongnyun Church. Thereafter, the patient stayed home after becoming symptomatic on January 30, 2020. Following her identification as a contact of confirmed patient #6 on January 31, 2020, she was quarantined in her home. During quarantine, on February 5, 2020, the patient visited a screening clinic of public health center in Seongbuk-gu, Seoul with a complaint of sore throat and was tested for 2019-nCoV disease. The patient was confirmed with the infection on the same day and was transferred to the Seoul National University Hospital for isolation.

(Contacts of patient #21 and preventive measures for them)

As of February 7, 2020, a total of 7 people had come in contact with patient #21 and are currently being monitored after being quarantined at their respective homes.

(Transmission of infection by patient #21)

As of February 7, 2020, infection by patient #21 has not been reported.

Case #22. (1st generation patient, Korea, infected by patient #16)

(Clinical progression of patient #22)

Patient #22 is a 46-year-old male of Korean nationality, and is a family member (older brother) of patient #16. Patient #22 was tested for 2019-nCoV disease while under home quarantine, after patient #16 was confirmed with the infection, on February 4, 2020, and positive result was obtained on February 6, 2020 at 1:00 a.m. The patient was then isolated in the Chosun University Hospital. Patient #22 reportedly had lunch with 7 family-members including patients #16 and #18 on January 25, 2020. The patient has not developed any symptoms as of February 7, 2020, and the case appears to be that of an asymptomatic infection. Patient #22 was discharged on February 15, 2020 after he was be completely cured.

(Contacts of patient #22 and preventive measures for them)

As of February 7, 2020, 1 person (his wife) has been identified as a contact of patient #22, and she tested negative. Patient #22 visited his workplace and a mall before he was diagnosed with the infection on February 6, 2020 at 1:00 a.m. However, as the patient was asymptomatic, only one of his co-workers has been quarantined at home, while no such action has been taken for the people who came into contact with him at the mall.

(Transmission of infection by patient #22)

As of February 7, 2020, infection by patient #22 has not been reported.

Case #23. (Index case, China)

(Clinical progression of patient #23)

Patient #23 is a 57-year-old female of Chinese nationality. The patient arrived on January 23, 2020, in Korea, planning to go on a group-tour and visit her daughter who is studying in Korea. The patient had no respiratory symptoms such as fever and cough at the time of arrival. Although her fellow travelers were subjected to total examination as they had come from Wuhan, their stay at Korea was extended as Wuhan city was closed-off. As a result, they had to move out from the original hotels that were reported on their disembarkation cards. Therefore, they could not be located by the quarantine authority. Patient #23 became symptomatic on February 3, 2020 and was found on February 4, 2020 during the process of locating and monitoring foreign visitors in the city of Seoul, with help from police. On February 5, 2020, the patient was tested for 2019-nCoV disease at the Seodaemun-gu public health center and was confirmed with the infection on February 6, 2020. She was isolated in the National Medical Center.

(Contacts of patient #23 and preventive measures for them)

Totally, 7 people who traveled with patient #23 tested negative. They are currently under home-quarantine at the multi-household residence at which they are staying.

(Transmission of infection by patient #23)

As of February 7, 2020, infection by patient #23 has not been reported.

Case #24. (Index case, Korea)

(Clinical progression of patient #24)

Patient #24 is a 28-year-old male of Korean nationality. As one of the first round group of 368 Koreans who were evacuated from Wuhan on January 31, 2020, the patient was quarantined at the Asan Police Human Resources Development Institute. He tested negative for 2019-nCoV disease at the time of arrival at the facility. However, during his quarantine at the facility, the patient developed symptoms of sore throat on February 6, 2020 and was re-tested. As the result was positive, the patient was isolated in the National Medical Center at 9:00 p.m., on the same day. Patient #24 is a co-worker of patient #13, who traveled with the former, to Wuhan for business and was diagnosed with 2019-nCoV disease on February 2, 2020. Both shared a room during the business trip to Wuhan, travelled to Korea on the same chartered flight on January 31, 2020 and were transferred to the Asan Police Human Resources Development Institute in the same bus.

(Contacts of patient #24 and preventive measures for them)

No one came into contact with patient #24 as he was quarantined on arrival in a single-bed room in the designated facility. However, as of February 7, 2020, additional tests are being conducted on the Koreans who took the same bus as patient #24, on January 31, 2020. Furthermore, two of the patient's co-workers who have been quarantined in the same Asan facility, along with other business employees who had worked with them in China and are now in the same facility, will also be tested.

(Transmission of infection by patient #24)

Epidemiologic investigation is currently ongoing, as of February 7, 2020.

Case #25. (1st generation patient, Korea, infected by patient #27)

(Clinical progression of patient #25)

Patient #25 is a 73-year-old female of Korean nationality. She experienced fever, cough, and sore throat on February 6, 2020, and visited the Sincheon Allied Hospital selective screening clinic in Siheung on February 7, 2020. At the time, # 25 had no history of visiting China, and son and daughter-in-law who lives with the patient visited China. However, The #25 was not included in the test subject because son and daugther-in-law were not visitors to Wuhan. On February 8, 2020 the symptoms did not improve, so she revisited Siheung Shinchon Allied Hospital and conducted a test. She was confirmed with the infection on February 9, 2020 and was isolated at Seoul National University Bundang Hospital. The patient had no visit to China, but her son and daughter-in-law returned home on January 31, 2020 after visiting Guangdong, China.

(Contacts of patient #25 and preventive measures for them)

Total of 11 people had come in contacts with patient #25 between February 5, 2020, one day before the onset of symptoms, and February 9, 2020 and are currently being monitored after being quarantined at their respective homes.

(Transmission of infection by patient #25)

As of February 11, 2020, infection by patient #25 has not been reported.

Case #26. (1st generation patient, Korea, infected by patient #27), Case #27. (Index case, China)

(Clinical progression of patient #26, #27)

Patient #26 is a 51-year-old male of Korean nationality and patient #27 is a 37-year-old female of Chinese nationality. They are husband and wife and stayed together in same place with patient #25. Patients # 26 and # 27 entered the Incheon Airport via Macau on the 31st of January on flight NX826, and had no fever at the time of entry. The patient #27 developed cough since January 24, 2020 while in China. The patient #27 developed fever, cough, and sore throat on February 5, 2020 and visited Sincheon Allied Hospital selective screening clinic in Siheung. But she was tested for influenza and chest X-ray, not 2019-nCoV disease test and the test was negative. So she went back home. Patient # 27 developed sore throat on February 8, 2020. On February 9, 2020, patient #25 living together was confirmed with the infection, and # 26 and # 27 patients were classified as close contacts of patient #25 and isolated in Anseong Hospital of Gyeonggi-do Medical Center. On the same day, patients #26 and #27 were confirmed with the infection. Patient #26 and #27 do trade busi-

ness and did not visit Wuhan, Hubei, China recently. When they stay in Guangdong, they did not visit hospital or market and they did not eat wild animals. They met Chinese people, however, they said they do not remember any contact with confirmed patient with 2019-nCoV disease.

(Contacts of patient #26, #27 and preventive measures for them)

Total of 38 people had come in contact with patient #26 and #27 and are currently being monitored after being quarantined at their respective homes.

(Transmission of infection by patient #26, #27)

Patients # 25 and # 26 were infected by patient # 27 and classified as first-generation patients.

Case #28. (1st generation patient, China, infected by patient #3)

(Clinical progression of patient #28)

Patient #28 is a 30-year-old female of Chinese nationality and entered Incheon Airport from Wuhan, China on January 20, 2020 with patient #3. Since entering Korea, she was classified as a close contact of patient #3 and has been quarantined at home since January 26, 2020. On February 8, 2020 near the completion of incubation period, she got tested for 2019-nCoV disease and the result was on the borderline between positive and negative. Two times of retests were conducted on February 9, 2020 and 10, 2020, and finally on February 10, 2020, the result was positive and isolated in Myongji Hospital. Patient #28 did not develop fever during home quarantine and she was taking painkiller, which was prescribed for one week from plastic surgery clinic where she visited with patient #3 before quarantine. So it is not easy to estimate the date of symptom-onset for her.

(Contacts of patient #28 and preventive measures for them)

Patient # 28 was quarantined at home in the patient #3's home until the confirmation of 2019-nCoV disease on February 10, 2020, and one contact, the mother of patient # 3 who lived with patient #28 during quarantine tested negative.

(Transmission of infection by patient #28)

As of February 10, 2020, infection by patient #28 has not been reported.

REFERENCES

A1. Wu JT, Leung K, Leung GM. Nowcasting and forecasting the potential domestic and international spread of the 2019-nCoV outbreak originating in Wuhan, China: a modelling study. Lancet 2020. doi: https://doi.org/10.1016/S0140-6736(20)30260-9S0140-6736(20)30260-9.
